# The 15q11.2 BP1-BP2 Microdeletion (*Burnside–Butler*) Syndrome: In Silico Analyses of the Four Coding Genes Reveal Functional Associations with Neurodevelopmental Disorders

**DOI:** 10.3390/ijms21093296

**Published:** 2020-05-06

**Authors:** Syed K. Rafi, Merlin G. Butler

**Affiliations:** Departments of Psychiatry & Behavioral Sciences and Pediatrics, University of Kansas Medical Center, Kansas City, KS 66160, USA

**Keywords:** 15q11.2 BP1-BP2 deletion (*Burnside–Butler*) syndrome, *NIPA1*, *NIPA2*, *CYFIP1*, *TUBGCP5*, Gene Ontology (GO), biological processes, gene interactions and pathways, associated diseases, neurodevelopmental disorders, autism

## Abstract

The 15q11.2 BP1-BP2 microdeletion (*Burnside–Butler*) syndrome is emerging as the most frequent pathogenic copy number variation (CNV) in humans associated with neurodevelopmental disorders with changes in brain morphology, behavior, and cognition. In this study, we explored functions and interactions of the four protein-coding genes in this region, namely *NIPA1*, *NIPA2*, *CYFIP1*, and *TUBGCP5*, and elucidate their role, in solo and in concert, in the causation of neurodevelopmental disorders. First, we investigated the STRING protein-protein interactions encompassing all four genes and ascertained their predicted Gene Ontology (GO) functions, such as biological processes involved in their interactions, pathways and molecular functions. These include magnesium ion transport molecular function, regulation of axonogenesis and axon extension, regulation and production of bone morphogenetic protein and regulation of cellular growth and development. We gathered a list of significantly associated cardinal maladies for each gene from searchable genomic disease websites, namely MalaCards.org: HGMD, OMIM, ClinVar, GTR, Orphanet, DISEASES, Novoseek, and GeneCards.org. Through tabulations of such disease data, we ascertained the cardinal disease association of each gene, as well as their expanded putative disease associations. This enabled further tabulation of disease data to ascertain the role of each gene in the top ten overlapping significant neurodevelopmental disorders among the disease association data sets: (1) Prader–Willi Syndrome (PWS); (2) Angelman Syndrome (AS); (3) 15q11.2 Deletion Syndrome with Attention Deficit Hyperactive Disorder & Learning Disability; (4) Autism Spectrum Disorder (ASD); (5) Schizophrenia; (6) Epilepsy; (7) Down Syndrome; (8) Microcephaly; (9) Developmental Disorder, and (10) Peripheral Nervous System Disease. The cardinal disease associations for each of the four contiguous 15q11.2 BP1-BP2 genes are *NIPA1*- Spastic Paraplegia 6; *NIPA2*—Angelman Syndrome and Prader–Willi Syndrome; *CYFIP1*—Fragile X Syndrome and Autism; *TUBGCP5*—Prader–Willi Syndrome. The four genes are individually associated with PWS, ASD, schizophrenia, epilepsy, and Down syndrome. Except for *TUBGCP5*, the other three genes are associated with AS. Unlike the other genes, *TUBGCP5* is also not associated with attention deficit hyperactivity disorder and learning disability, developmental disorder, or peripheral nervous system disease. *CYFIP1* was the only gene not associated with microcephaly but was the only gene associated with developmental disorders. Collectively, all four genes were associated with up to three-fourths of the ten overlapping neurodevelopmental disorders and are deleted in this most prevalent known pathogenic copy number variation now recognized among humans with these clinical findings.

## 1. Introduction

The 15q11.2 BP1-BP2 deletion (*Burnside–Butler*) syndrome is an emerging condition that encompasses four protein-coding genes (*NIPA1, NIPA2, CYFIP1,* and *TUBGCP5*) within this chromosome region. When disturbed, these four genes lead to cognitive impairment with speech and/or motor delay along with dyslexia and psychiatric/behavior problems (attention deficit hyperactivity, autism, schizophrenia or psychosis), ataxia or poor coordination, seizures, congenital anomalies and structural brain defects [1]. These genes are associated with neurological, cognitive, or behavior problems as well as playing a role in both Prader–Willi and Angelman syndromes, first examples in humans of genomic imprinting [2].

These imprinting disorders typically are caused by a deletion in the majority of cases involving the 15q11-q13 chromosome region of different parental origin [i.e., paternal in Prader–Willi syndrome (PWS) and maternal in Angelman syndrome (AS)] [1,2]. The typical 15q11-q13 deletion involves proximal chromosome 15 breakpoints BP1 or BP2 and the more distally placed BP3 containing repetitive DNA segments allowing malalignment in meiosis of the homologous chromosome 15s leading to deletions or duplications in the region. The individuals with the larger typical type I deletion (involving BP1 and BP3) are found in both Prader–Willi and Angelman syndromes. They are reported with more severe developmental symptoms and clinical severity than individuals with the smaller typical type II deletion (involving BP2 and BP3) [3]. The 15q11.2 BP1-BP2 microdeletion encompasses a 500kb region located between breakpoints BP1 and BP2 proximal to the centromere of chromosome 15 including the four protein-coding genes (i.e., *NIPA1*, *NIPA2*, *CYFIP1*, *TUBGCP5*) that become the major focus of this review.

Hundreds of patients have now been reported with the 15q11.2 BP1-BP2 microdeletion often associated with the above listed neurodevelopmental disorders. A summary of the clinical features reported in over 200 individuals were described by Cox and Butler [4] and further reviewed by Butler [1] by grouping into five categories. These categories are (1) growth and development; (2) dysmorphic features; (3) intelligence and academic achievement; (4) behavioral and psychiatric problems, and (5) other related medical concerns. Developmental problems were reported in 73% of cases and speech delay in 67%; dysmorphic ears (46%) and palatal anomalies (46%); writing (60%) and reading (57%) difficulties, memory problems (60%) and verbal IQ scores ≤75 (50%); behavior problems, unspecified (55%); and abnormal brain imaging findings (43%). Additional clinical features were not as common and included motor delay (42%), ADD/attention deficit hyperactivity disorder (35%), autism spectrum disorder (27%), seizures or epilepsy (26%), and schizophrenia/paranoid psychosis (20%).

A recent report on structural brain anomalies using detailed imaging in hundreds of patients with the chromosome 15q11.2 BP1-BP2 microdeletion and controls showed a smaller brain surface area with a thicker cortex. These findings were more common in the frontal, cingulate, and parietal lobes in those with the deletions, supporting clinical observations and evidence as an emerging syndrome. Aberrations, leading to copy number variation (CNV) in these genes are estimated to be present in 0.5% to 1.0% of the population, making this specific site as the most prevalent known pathogenic copy number variation in humans [5]. However, not all individuals with this microdeletion are clinically affected, yet the collection of findings appears to share biological pathways that are hitherto unexplored. Their presumed genetic mechanisms require further analysis, as illustrated and discussed in our report.

Summarized results from chromosomal microarray analysis by a certified commercial clinical testing laboratory of patients presenting with genetic services were reported by Ho et al. [6]. The microarray analysis included 2.8 million probes optimized for the detection of CNVs associated with neurodevelopmental disorders. They reported an overall CNV detection rate of 28.1% in 10,351 consecutive patients which rose to nearly 33% of cases without ASD but with developmental delay/intellectual disability and/or multiple congenital anomalies. The overall rate of detection for those with ASD was also significant at 24.4%. The 15q11.2 BP1-BP2 deletion (*Burnside–Butler*) syndrome was found to be the most common disturbance (9%) seen in 85 genetic defects associated with neurodevelopmental disorders in this large cohort of consecutive patients and the most common finding in those presenting with ASD.

**Figure 1:**
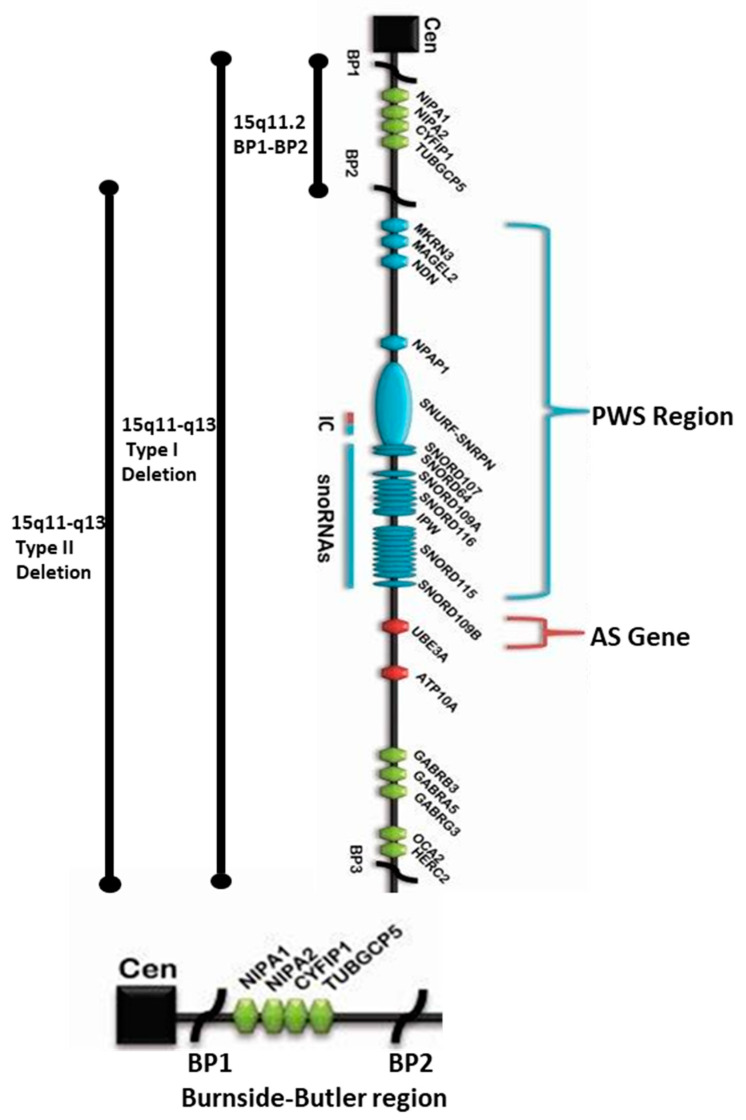
15q11.2 BP1-BP2 microdeletion (*Burnside–Butler*) syndrome region found at the proximal end of Prader–Willi syndrome (PWS) / Angelman syndrome (AS) regions within the 15q11-q13 Type I deletion depicting the location and order of the four protein-coding genes therein: *NIPA1*, *NIPA2*, *CYFIP1*, and *TUBGCP5* within the 15q11.2 region distal to the centromere and proximal to the imprinted PWS/AS genes. The bottom enlarged horizontal chromatin figure exclusively depicts *NIPA1*, *NIPA2*, *CYFIP1*, and *TUBGCP5* genes in the BP1-BP2 region.

The larger 15q11-q13 type I deletion is approximately 6.6Mb in size seen in both PWS and AS and includes four genes (*NIPA1*, *NIPA2*, *CYFIP1*, and *TUBGCP5*) located within the proximal 15q11.2 BP1-BP2 microdeletion region. The smaller typical 15q11-q13 type II deletion has these four genes intact (see Figure 1). These genes are highly conserved and expressed in the brain (GeneCards.org & UniProtKB/Swiss-Prot). The *NIPA1* gene causes autosomal dominant hereditary spastic paraplegia and postural disturbances when disturbed (https://www.malacards.org/card/spastic_paraplegia_6_autosomal_dominant) and functions as a magnesium transporter (https://www.genecards.org/cgibin/carddisp.pl?gene=NIPA1&keywords=NIPA1).

Mutations of the NIPA2 gene are reported in patients with childhood absence epilepsy with decreased intracellular magnesium concentration in neurons [7,8,9,10,11]. The CYFIP1 gene encodes a protein product that interacts with FMRP, the protein coded by the FMR1 gene causing fragile X syndrome (Entrez Gene: 23191; https://www.genecards.org/cgi-bin/carddisp.pl?gene=CYFIP1&keywords=CYFIP1). The fourth gene is TUBGCP5 associated with the chromosome 15q11.2 deletion syndrome and obsessive-compulsive disorder when disturbed. It also plays a role in microtubule nucleation at the centrosome in cells (UniProtKB: Q96RT8; https://www.genecards.org/cgi-bin/carddisp.pl?gene=TUBGCP5&keywords=TUBGCP5) [8,9,10,11,12,13,14,15,16,17,18].

**Overview of 15q11-q13 BP1-BP3 Region Depicting the Proximal Location of BP1-BP2 Microdeletion (Burnside–Butler) Syndrome Region Within the Broader Type I Deletion Adjacent to Prader–Willi Syndrome (PWS)/Angelman Syndrome (AS) Regions.**

The location, as well as the order of genes and transcripts (e.g., snoRNAs), are also shown in Figure 1 above that causes PWS/AS. Those genes that are imprinted and paternally expressed causing PWS (in blue) and those causing AS (in red) are also imprinted and maternally expressed. The location and the relative size of the 15q11.2 BP1–BP2 microdeletion region, the typical larger 15q11–q13 Type I deletion involving breakpoints BP1 and BP3, and the typical smaller 15q11–q13 Type II deletion involving breakpoints BP2 and BP3 in both PWS and AS are illustrated. IC: imprinting center controlling the activity of imprinted genes in the 15q11–q13 region.

## 2. Results

### 2.1. Overview of the Four Genes in the 15q11.2 BP1-BP2 Region

**Figure 2:**
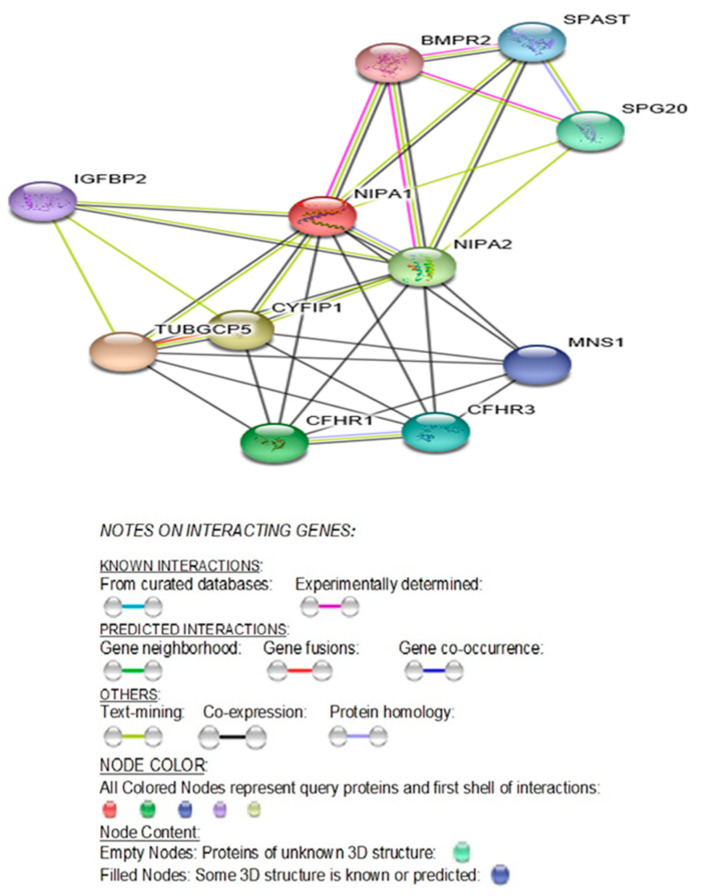
STRING Protein-Protein Interaction network involving *NIPA1*, *NIPA2*, *CYFIP1*, and *TUBGCP5* genes with functional interactions showing 11 nodes (Table 1) and 34 edges and predicted functional interactions, such as, biological process (GO) and molecular function (GO) (Table 2), as designated in STRING: 9606.ENSP00000337452 (*STRING Consortium 2019*). Network nodes represent proteins (Table 1) with splice isoforms or post-translational modifications collapsed into each node for all proteins produced by a single protein-coding gene. Edges represent protein-protein associations that are considered specific and meaningful, i.e., proteins jointly contribute to a shared function, such as, Biological Process (GO) and Molecular Function (GO) (Table 2).

All four syntenic and bi-allelically conserved expressed genes in the 15q11.2 region between breakpoints BP1 and BP2 are functionally predicted to interact with each other along with seven other genes. The predicted STRING functional interactions network encompasses these four genes as illustrated in Figure 2.

The STRING diagram and protein interactions involving all four genes of this microdeletion syndrome are depicted in Figure 2 and their predicted functions are presented in Table 1. The identified biological processes (GO) and molecular functions (GO) are presented in Table 2 (https://version11.stringdb.org/cgi/network.pl?taskId=XJOUKPc8icHM).

**Protein Network (STRING) Interactions Encompassing the Four Genes: *NIPA1*, *NIPA2*, *CYFIP1* and *TUBGCP5***

### 2.2. NIPA1 (Non-Imprinted in Prader–Willi/Angelman Syndrome Region Protein 1) Gene

Attributes, Location, Description, Function and Associated Disorders for NIPA1 Gene.

The chromosomal band location for the *NIPA1* gene is 15q11.2 with the genomic location (GRCh38/hg38) at 22,773,063–22,829,789 with a size of 56,727 bases in a plus-strand orientation. This gene has the following attributes: Size: 329 amino acids; Molecular mass: 34,562 Da; Quaternary structure:Homodimer (https://www.genecards.org/cgi-bin/carddisp.pl?gene=NIPA1&keywords=NIPA1). NIPA1 has two alternative splice isoforms; isoform 2 differs from the canonical sequence by missing the first 75 amino acids (GeneCards & UniProtKB/Swiss-Prot). It is a multi-pass membrane protein recruited to the cell membrane in response to low extracellular magnesium (GeneCards & UniProtKB/Swiss-Prot). This gene is widely expressed with highest levels in neuronal tissues and overexpressed in the spinal cord (20.8), frontal cortex (17.0), and fetal liver (7.3) (*Protein differential expression in normal tissues from Human Integrated Protein Expression Database (HIPED) for NIPA1 Gene*: https://www.genecards.org/Guide/GeneCard#protein-differential-expression; https://www.genecards.org/cgi-bin/carddisp.pl?gene=NIPA1&keywords=NIPA1).

NIPA1 protein plays a potential role in nervous system development and maintenance and is most ubiquitously expressed in the brain with a mean RPKM (*Reads Per Kilobase per Million reads placed*) of 16.56 + 3.055 (https://www.ncbi.nlm.nih.gov/gene/123606/?report=expression; https://www.gtexportal.org/home/gene/NIPA1) [14]. The multiple transmembrane domains of this protein localize to endosomes and plasma membrane. This protein is recruited in response to low extracellular magnesium and functions in Mg2 transport (Q7RTP0-NIPA1_HUMAN) [15]. Subcellular localization with immunofluorescence shows that endogenous *NIPA1* protein associates with early endosomes and the cell surface in a variety of neuronal and epithelial cells. As expected of a magnesium-responsive gene, altered magnesium concentration leads to redistribution between the endosomal compartment and the plasma membrane; high magnesium results in diminished cell surface *NIPA1* whereas low magnesium leads to accumulation in early endosomes and recruitment to the plasma membrane [15].

An important paralog of this gene, *NIPAL1* is also a magnesium ion transmembrane transporter. In addition, there are three other protein-coding transcript variants (paralogs): *NIPAL2, NIPAL3,* and *NIPAL4*, which are also magnesium ion transmembrane transporters. Other genes also perform a similar magnesium transport function, namely, *MAGT1*, *MMGT1*, and *MRS2* (GeneCards.Org). Although *NIPA1* protein acts as a Mg (2+) transporter, it can also transport other divalent cations such as Fe(2+), Sr(2+), Ba(2+), Mn(2+), and Co(2+), but to a much less extent than Mg(2+) (UniProtKB/Swiss-Prot Summary). Among its other related pathways are the transport of glucose and other sugars, bile salts and organic acids, metal ions, and amine compounds.

Only mutations (not haploinsufficiency due to 15q11.2 BP1-BP2 microdeletions including the *NIPA1* gene) have thus far been associated with autosomal dominant spastic paraplegia 6 / SPG6- linked hereditary spastic paraplegia: HSP (https://www.genecards.org/cgi-bin/carddisp.pl?gene=NIPA1&keywords=NIPA1). Spastic paraplegia 6 is a neurodegenerative disorder characterized by slow, gradual, progressive weakness and spasticity of the lower limbs. Rate of progression and the severity of symptoms are quite variable with initial symptoms of difficulty with balance, weakness, and stiffness in the legs, muscle spasms, and dragging the toes when walking. In some forms of the disorder, bladder symptoms (such as incontinence) may appear, or weakness and stiffness may be present to other parts of the body (UniProtKB/Swiss-Prot) [16,17,18]. Two variants in the *NIPA1* gene, namely, p.Thr45Arg (VAR_023440; SNP ID: rs104894496) and p.Gly106Arg (VAR_023441; SNP ID: rs104894490) are disease-causing variations for spastic paraplegia type 6 (SPG6), autosomal dominant form. A rare *NIPA1* deletion was also found in a patient with pervasive developmental disorder not otherwise specified and with mild intellectual disability [19]; this deletion is also linked to autism (SFARI.org).

The mouse NIPA1 mutants, p.Thr39Arg and p.Gly100Arg, corresponding to the respective human mutants are associated with hereditary spastic paraplegia (HSP) showing a loss-of-function when expressed in oocytes and altered trafficking in transfected COS7 cells [15]. The *NIPA1* gene normally encodes an Mg2+ transporter protein and the loss-of-function of *NIPA1*, due to abnormal trafficking of the mutated protein provides the basis for the HSP phenotype [15,16,20]. Only abnormal trafficking of the mutated protein is causative for HSP and not from its deficiency as evident by Prader–Willi syndrome (PWS) or Angelman syndrome (AS) with only one copy of the *NIPA1* gene in the typical 15q11-q13 type I deletion in both PWS or AS involving breakpoints BP1 and BP3 including the four genes in the 15q11.2 BP1-BP2 region. Other genes or transcripts, both imprinted and biallelic, do not have HSP [16,21]. Key findings in PWS and AS develop from errors of imprinting dependent on the parent of origin [22,23]. Therefore, SPG6 linked HSP is more likely caused by a dominant-negative effect or a toxic gain-of-function mechanism rather than a loss-of-function [16,17,21,24,25].

A perusal of genes involved in numerous other forms of spastic paraplegia indicates causation by genetic defects in various other metabolic pathways unrelated to magnesium transporter protein deficiency (see GeneCards.org). The syntenic *NIPA2* gene, which also codes a magnesium ion transmembrane transporter protein and along with *NIPA1* in the same region are both deleted in the Type I deletion but not deleted in the smaller typical 15q11-q13 Type II deletion in PWS or AS. However, patients with the larger Type I deletion do not have spastic paraplegia (GeneCards.org). Mutations in the *NIPA2* gene have been reported in generalized epilepsy and childhood absence epilepsy [10,11,26]. In addition to the magnesium ion transmembrane transporter activity, *NIPA1* protein inhibits Bone Morphogenetic Protein signaling by regulating the endosomal trafficking and degradation of type 2 BMP receptors (*BMPR2*) in Drosophila and HeLa cells [27,28].

Bone Morphogenetic Proteins (BMPs) are a group of signaling molecules that belong to the Transforming Growth Factor-β (*TGFβ*) superfamily of proteins. Initially discovered for their ability to induce bone formation, BMPs are now known to play crucial roles in all organ systems. BMPs are important in embryogenesis and development, and also in the maintenance of adult tissue homeostasis. Another relevant role of BMP in the neurological system is neurogenesis. Neural defects are associated with loss of BMP function in mouse models [29]. For instance, *BMP11* is involved in spinal cord neurogenesis and secreted from neurons themselves serving as an inhibitory signal in the generation of new neurons from progenitors in the olfactory epithelium [30].

In rodents, BMP signaling is upregulated following lesions of the corticospinal track and suppressing upregulation that promotes regrowth of axons [31]. Targeting specific BMP receptor subunits for therapeutic purposes may provide an approach for manipulating gliosis and enhancing functional outcomes after spinal cord injury [32]. Therefore, it is no surprise that BMP signaling cuts across all hereditary spastic paraplegia (HSP) categories and are widely implicated in neurodegenerative diseases [33]. Among the HSP proteins, *NIPA1* is best characterized mechanistically to inhibit BMP signaling [15]. In summary, these studies support abnormal BMP signaling in many cases probably resulting from abnormal BMP receptor trafficking and could be a unifying pathogenic mechanism for some forms of hereditary spastic paraplegia [34].

**Figure 3:**
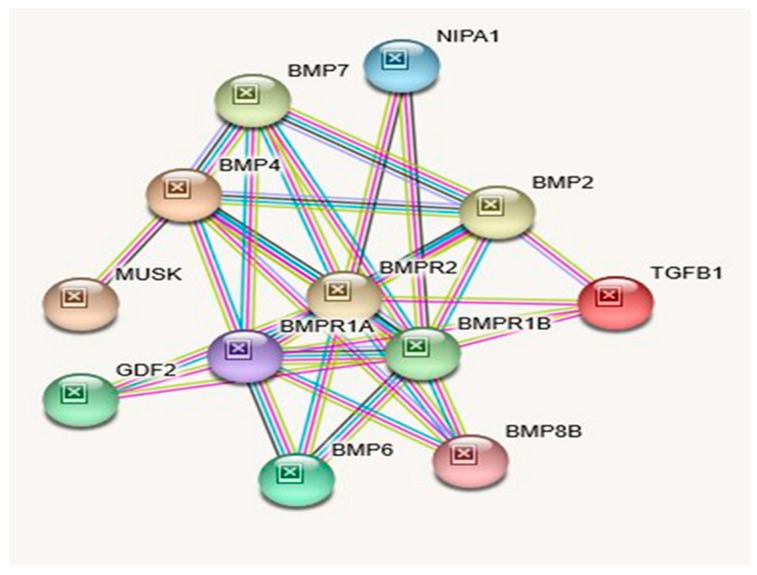
STRING Protein–Protein Interaction Network for *NIPA1* gene with functional interactions with 12 nodes (Source: STRING.org). Network nodes represent proteins with splice isoforms or post-translational modifications collapsed into each node for all proteins produced by a single protein-coding gene. Edges represent protein-protein associations that are considered specific and meaningful (i.e., proteins jointly contribute to a shared function, such as, Biological Process (GO) and Molecular Function (GO) (Table 3). STRING interactants and their functions related to other proteins associated with *NIPA1* gene are listed in Table 5. (See Figure 2 for legend with a description of symbols and notes on interpreting the interaction of genes and their encoded proteins).

Further highlighting the importance of BMP regulation in neural development is the role of *BMP7* in corticogenesis; *BMP7* deletions result in reduced cortical thickening and impaired neurogenesis [35]. Interestingly, BMPR type 1A (*BMPR1A*) is important in the establishment of neurons involved in regulating feeding behavior [32]. Similarly, *NIPA1* was shown to inhibit BMP signaling by regulating the endosomal trafficking and degradation of type 2 BMP receptors (*BMPR2*) in Drosophila and HeLa cells [27,28]. Perhaps, haploinsufficiency of *NIPA1* protein in cases of 15q11-q13 BP1 or BP2/BP3 deletions might partially affect its inhibition of BMP signaling by regulating endosomal trafficking and degradation of type 2 BMP receptors, thereby affecting neurons. This might be the causal factor for the reported developmental and language delay, neurobehavioral disturbances, and psychiatric problems, such as autism, seizures, and schizophrenia with occasional mild dysmorphic features seen to a varying degree in patients with the 15q11.2 BP1-BP2 deletion (*Burnside–Butler*) syndrome [1,36]. Biological processes (GO), molecular functions (GO), cellular components with KEGG, and Reactome pathways are summarized in Table 3. In Table 4 examples of diseases or disorders which have been recognized and associated with the *NIPA1* gene when disturbed are collated and ranked by order using MalaCards (MalaCards.org) from GeneCards (GeneCards.org). STRING functional interactions with nodes and edges (see Figure 3). Further illustrations of interactions and functions of NIPA1 with relationship to other proteins can be found in Table Table 5.

**Interacting Proteins for NIPA1 Gene: STRING Interaction Network.**

## 2.3. NIPA2 (Non-Imprinted in Prader–Willi/Angelman Syndrome Region Protein 2) Gene

**Attributes, Location, Description, Function, and Associated Disorders for NIPA2 Gene.**

The chromosomal band location for the *NIPA2* gene is 15q11.2 with genomic location (GRCh38/hg38) at 22,838,641- 22,868,384 with a size of 29,744 bases in a plus-strand orientation. This gene is protein-coding specifically for NIPA magnesium transporter 2 (https://www.genecards.org/cgi-bin/carddisp.pl?gene=NIPA2&keywords=nipa2). When disturbed (deleted), this gene is associated with both Angelman and Prader–Willi syndromes due to specific parent of origin deletions of the 15q11-q13 region. The typical 15q11-q13 type I deletion also includes the other three protein-coding genes in the 15q11.2 BP1-BP2 region, namely, *NIPA1, CYFIP1,* and *TUBGCP5*. In the case of the smaller typical 15q11-q13 type II deletion, these four genes remain intact in the region (Figure 1) [1,2,3].

The NIPA2 protein has the following attributes: Size: 360 amino acids; Molecular mass:39185 Da. Quaternary structure: No Data Available. This protein has two isoforms; isoform 2 differs from the canonical sequence as follows: 47–66: GQGGHAYLKEWLWWAGLLSM → V. Similar to NIPA1, NIPA2 is also a multi-pass membrane protein found in early endosomes recruited to the cell membrane in response to low extracellular magnesium (UniProtKB/Swiss-Prot for *NIPA2* Gene) (Table 6).

Unlike *NIPA1,* this gene is overexpressed in B-lymphocytes and the placenta ((UniProtKB/Swiss-Prot). In addition to PWS and AS involvement for this gene, it is also associated with childhood absence epilepsy [26], childhood electroclinical syndrome [10,11], and possibly autosomal recessive congenital ichthyosis (MalaCards/GeneCards) and important biological processes and functions (see Table 7).

Among related pathways of this gene are miscellaneous transport and binding events and transport of glucose and other sugars, bile salts and organic acids, metal ions and amine compounds. Gene Ontology (GO) annotations related to this gene also include magnesium ion transmembrane transporter activity. An important paralog of this gene is *NIPAL1* (GeneCards). Chai et al. [9] determined that the *NIPA2* gene contains 7 exons and spans 29 kb. The coding region extends between exons 3 and 7, and alternative splicing utilizes alternate exons 2 and 2b which results in multiple transcript variants. Pseudogenes of this gene are found on chromosomes 3, 7, and 21 (Entrez Gene: 81614).

**Figure 4:**
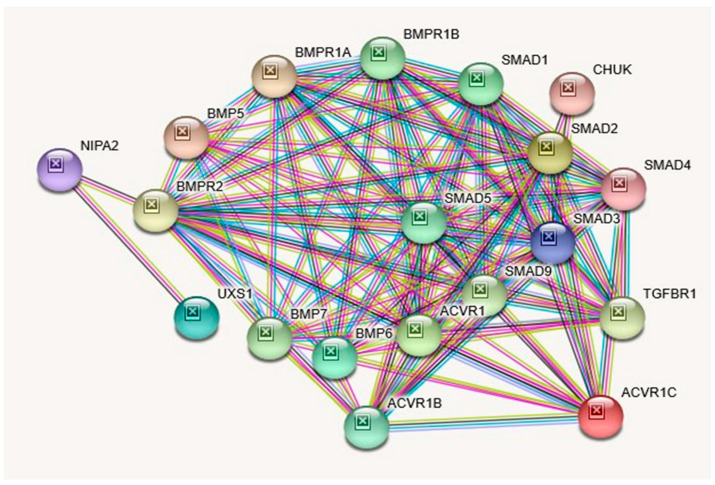
STRING Protein–Protein Interaction Network for *NIPA2* gene with functional interactions with 17 nodes. (Source: STRING.org). Network nodes represent proteins with splice isoforms or post-translational modifications collapsed into each node for all proteins produced by a single protein-coding gene. Edges represent protein-protein associations that are considered specific and meaningful (i.e., proteins jointly contribute to a shared function, such as, Biological Process (GO) and Molecular Function (GO) (Table 6). NIPA2 gene protein Interactants and their functions are listed in Table 8. (See Figure 2 for legend with a description of symbols and notes on interpreting the interaction of genes and their encoded proteins). STRING Protein Interactants and Their Functions for *NIPA2* gene are listed in Table 8.

Similar to *NIPA1*, this multi-pass cell membrane protein is localized in the plasma membrane and early endosomes (Gene Ontology (GO)—GO:0005769; GO:0005886) and recruited to the cell membrane in response to low extracellular magnesium (GeneCards; ECO:0000250). Paralogs for the *NIPA2* gene are *NIPA1, NIPAL1, NIPAL4, NIPAL2,* and *NIPAL3* (PMID: 28514442; [35]). Although *NIPA2* is highly conserved (PMID: 14508708; [9]) and expressed in parts of the brain as well as in most other organ systems, it is significantly overexpressed in B-lymphocytes and the placenta. In addition, five variants have been reported for this gene with either loss or gain of function or both (GeneCards). Table 8 describes the STRING protein interactions and their functions with this gene and description while Table 6 shows biological processes, molecular functions, cellular components, and KEGG and Reactome pathways attributed to the *NIPA2* gene. To examine NIPA2 protein interactions and connectivity with other genes and their interrelated proteins, see the STRING interaction network displayed in Figure 4.

**Protein–Protein Inter-Relationships Involving the NIPA2 Gene.**

## 2.4. CYFIP1 (Cytoplasmic FMR1 Interacting Protein 1) Gene

**Attributes, Location, Description, Function, and Associated Disorders for CYFIP1 Gene.**

The chromosomal band location for the *CYFIP1* gene is 15q11.2 with the genomic location (GRCh38/hg38) at 22,867,052–22,981,063 with a size of 114,012 bases in a minus strand orientation (Genecards.org). The CYFIP1 protein has the following attributes: Size: 1253 amino acids; Molecular mass: 145,182 Da. Quaternary structure: Component of the WAVE1 complex composed of ABI2, CYFIP1 or CYFIP2, BRK1, NCKAP1, and WASF1/WAVE1. CYFIP1 and CYFIP2 are part of the Wiskott–Aldrich syndrome protein-family verprolin-homologous protein (WAVE) complex that regulates actin polymerization at synapses and CYFIP1 protein has 3 described isoforms produced by alternative splicing (https://www.uniprot.org/uniprot/Q7L576) (see Table 9). This protein is required for neuronal and bristle development in *Drosophila* [37]. Through the CYFIP1 protein, the fragile X syndrome protein represses activity-dependent translation, and thus implicated in fragile X syndrome [38].

**Figure 5:**
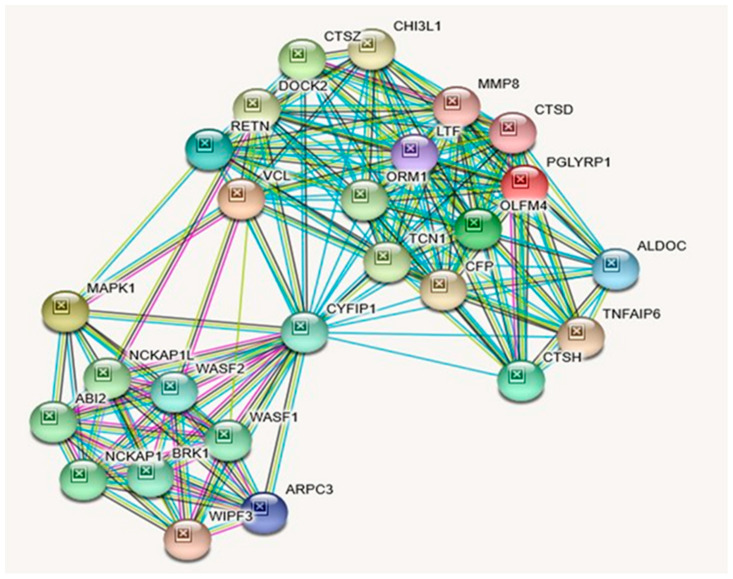
STRING Protein–Protein Interaction Network for *CYFIP1* gene with functional interactions with 26 nodes. (Source: STRING.org). Network nodes represent proteins with splice isoforms or post-translational modifications collapsed into each node for all proteins produced by a single protein-coding gene. Edges represent protein-protein associations that are considered specific and meaningful, i.e., proteins jointly contribute to a shared function, such as, Biological Process (GO) and Molecular Function (GO) (Table 9). *CYFIP1* gene protein–protein interactants and their functions are listed in Table 11. (See Figure 2 for legend with a description of symbols and notes on interpreting the interaction of genes and their encoded proteins).

The *CYFIP1* gene is highly expressed in the perinuclear region and enriched in synaptosomes. It is also enriched in membrane ruffles and at the tips of lamellipodia with the following subcellular localizations (GeneCards & UniProtKB/Swiss-Prot). The *CYFIP1* gene is widely expressed in all tissues (GenecCards). In addition to this gene’s association with fragile X syndrome, it is also associated with autism (see Table 10). A large chromosomal deletion including this gene is associated with increased risk of schizophrenia and epilepsy in human patients and reduced expression of this gene has also been observed in various human cancers, the encoded protein may inhibit tumor invasion (Entrez Gene summary: RefSeq, May 2017). Among this gene’s related pathways are the regulation of actin dynamics for phagocytic cup formation and signaling by Rho GTPases (Table 9). Gene Ontology (GO) annotations related to this gene include Rac GTPase binding. An important paralog of this gene is *CYFIP2* (GeneCards). Table 10 lists putative associated diseases for *CYFIP1* Gene. STRING protein interactions with their functions and descriptions related to *CYFIP1* are presented in Table 11. An illustration of the identified interactions can also be visualized in Figure 5.

Protein–Protein Inter-Relationships Involving CYFIP1 Gene.

## 2.5. TUBGCP5 (Tubulin Gamma Complex Associated Protein 5) Gene

**Attributes, Location, Description, Function, and Associated Disorders for TUBGCP5 Gene.**

The chromosomal band location for *TUBGCP5* gene is 15q11.2 with the genomic location (GRCh38/hg38) at 22,983,192–23,039,673 with a size of 56,482 bases in a minus strand orientation (Genecards.org). TUBGCP5 protein has the following attributes: Size: 1024 amino acids; Molecular mass:118321 Da; Quaternary structure: Gamma-tubulin complex which is composed of gamma-tubulin, *TUBGCP2, TUBGCP3, TUBGCP4, TUBGCP5* and *TUBGCP6* *proteins* (GeneCards & UniProtKB/Swiss-Prot). This protein has 2 isoforms produced by alternative splicing; the sequence of this isoform differs from the canonical sequence as follows: 1010–1024: LESLALSLMAGMEQS → CEYIMLKYFYLCISL. *TUBGCP5* gene is widely expressed with the highest levels in the heart and skeletal muscle with moderate levels in the brain (GeneCards & UniProtKB/Swiss-Prot). An important paralog of this gene is *TUBGCP6*.

**Figure 6:**
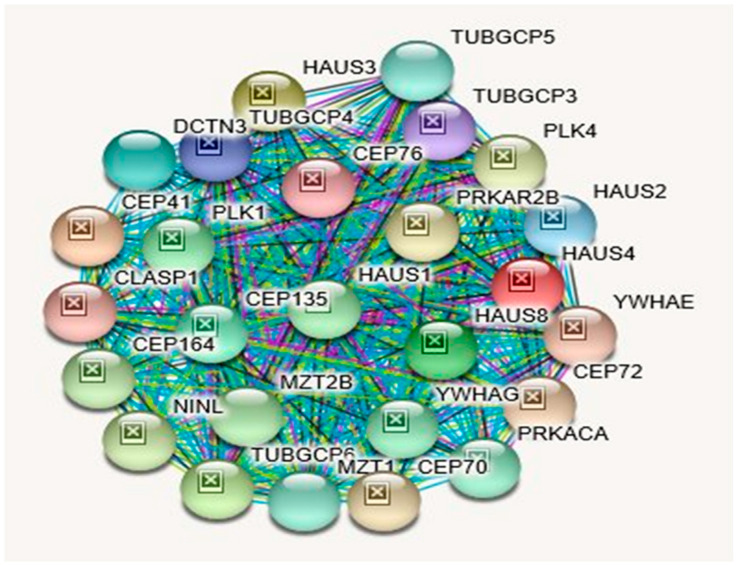
STRING Protein–Protein Interaction Network for *TUBGCP5* gene with functional interactions with 26 nodes (Source: STRING.org). Network nodes represent proteins with splice isoforms or post-translational modifications collapsed into each node for all proteins produced by a single protein-coding gene. Edges represent protein-protein associations that are considered specific and meaningful i.e., proteins jointly contribute to a shared function, such as, Biological Process (GO) and Molecular Function (GO) (Table 12). *TUBGCP5* gene interactants and their functions are listed in Table 14. (See Figure 2 for legend with a description of symbols and notes on interpreting the interaction of genes and their encoded proteins).

Table 12 shows biological processes, molecular functions, cellular components with KEGG and Reactome pathways related to TUBGCP5. Among its related pathways are Nanog in Mammalian ESC Pluripotency and G-Beta Gamma Signaling. Gene Ontology (GO) annotations related to this gene include microtubule binding, and components of the cytoskeleton (Table 12). Table 13 shows diseases associated with *TUBGCP5* gene disturbances based on MalaCards and arranged in descending order including Prader–Willi syndrome as the top disorder followed by schizophrenia and then autism. Table 13 shows proteins and their functions that interact with *TUBGCP5*, as depicted in Figure 6, illustrating the interactions of the *TUBGCP5* gene with other related genes.

**Protein–Protein Inter-Relationships Involving the TUBGCP5 Gene.**

## 3. Discussion

Features seen in patients with the 15q11.2 BP1-BP2 deletion (*Burnside–Butler*) syndrome can be quite variable. Different clinical phenotypes are seen in children with this disorder and can depend on the source of the parental deletion [36]. Recent evidence of partial expression or bias of these four genes depending on the parent of origin leads to possible clinical differences in the offspring. For example, when the deletion is paternal, there is a greater risk of having congenital heart defects [36]. If the mother transmits the deletion, then there is a greater risk of intellectual disability and autism in their affected children [36]. A recent review further showed a phenotype with global and regional measures of surface area and cortical thickness in the brain with subcortical volumes and cognition differences compared to controls. This investigation consisted of 203 individuals compared with 4500 controls without the deletion collected from years 2015–2019 and the average age was 56 years [5]. This international study analyzed the largest CNV and neuroimaging results to date in those with the 15q11.2 BP1-BP2 deletions and non-deletion controls. They reported reduced brain surface area, a thicker cortex, and a smaller nucleus accumbens in the 15q11.2 BP1-BP2 deletion subjects. The significant difference in cortical thickness was more evident in the frontal, cingulate and parietal lobes. Furthermore, cognitive ability was lower for those with the deletion compared with non-deletion individuals suggesting involvement in neural plasticity and development leading to functional brain differences identified clinically [5].

As noted in Table 15, all four genes are protein-coding genes and neither were maternally nor paternally imprinted. Their encoded proteins interact with each other in crucial biological processes and molecular pathways (Figure 2, Table 1 and Table 2). The predicted functional interactions encompassing all four genes included 11 nodes with 34 edges that pertain to biological processes and molecular functions utilizing searchable genomic databases involving the four genes in this 15q11.2 BP1-BP2 microdeletion syndrome (see Table 1). Among them, in addition to magnesium ion transport molecular function, significant other biological processes, such as regulation of axonogenesis and axon extension, regulation and production of bone morphogenetic protein (BMP), regulation of cellular growth and development were observed. These biological processes are relevant to variable clinical phenotypes, such as, autism, seizures, and schizophrenia which are also seen in this microdeletion syndrome. These clinical phenotypes often affect neurological development and are occasionally accompanied by mild dysmorphic features [1,9,10,11,12,13].

Although *NIPA1* and *NIPA2* share related KEGG and Reactome pathways (Table 3 and Table 6), their cardinal disease associations are different (Table 15): Spastic Paraplegia 6, Autosomal Dominant and Spastic Paraplegia 6 for *NIPA1* and Angelman Syndrome and Prader–Willi Syndrome for *NIPA2*. However, as shown in Table 16, they both are also associated with PWS and AS. Unlike *NIPA1* and *NIPA2, CYFIP1*, and *TUBGCP5* genes do not share any KEGG or Reactome pathways (Table 9 and Table 12). As shown in Table 15, cardinal diseases that are associated with the *CYFIP1* gene are Fragile X Syndrome and Autism. The cardinal disease that is associated with the *TUBGCP5* gene is Prader–Willi syndrome. However, as depicted in Table 16, they both are additionally associated with PWS, while the *CYFIP1* gene alone is additionally associated with AS. Most notably, all four syntenic genes in this region are associated with Autism Spectrum Disorder; Schizophrenia; Epilepsy and Down Syndrome (Table 16). Except for *TUBGC5*, all three genes are associated with the 15q11.2 BP1-BP2 Deletion Syndrome with Attention Deficit Hyperactive Disorder & Learning Disability (Table 16). It should be noted that interacting proteins for CYFIP1 and TUBGCP5 proteins are quite different (Figure 5, Table 11; Figure 6, Table 14, respectively), which is unlike the case of *NIPA1* and *NIPA2* (Figure 3, Table 5; Figure 4, Table 8, respectively).

It is interesting to note that none of the protein-protein interacting genes for the four genes, which total 70, are the same (Table 5, Table 8, Table 11, and Table 14). However, NIPA1 and NIPA2 proteins, given their involvement with common biological processes (GO) and molecular functions (GO) along with BMP6, BMP7, BMPR1A, BMPR1B, and BMPR2-proteins interact with both. In contrast, none of the protein-protein interactants are similar between CYFIP1 and TUBGCP5 proteins (Table 11 and Table 14), thus affirming their functional dissimilarities.

It should be noted, as illustrated in STRING Figure 2 and Table 1, NIPA1, NIPA2, CYFIP1, and TUBGCP5 proteins show protein–protein interactions among themselves with the 11 nodes and 34 edges addressed earlier. As shown in Table 1, NIPA1 protein interacts with TUBGCP5, CYFIP1, and NIPA2 proteins (scores: 0.995, 0.967, and 0.941, respectively) along with seven other protein interactants, and thus predicted to cause significant biological processes (Table 2), and a significant molecular function is magnesium ion transmembrane transporter activity with a 0.0042 false discovery rate (Table 2).

The varied biological processes and predicted functions of these four genes, as noted above, through their protein–protein interactions with seven other proteins (see Figure 2) collectively could play a role in the neurodevelopmental disorders. They complement regulation associated with lipoproteins and lipid metabolism and adipogenesis, encompassing CFHR1, CFHR3, and BMPR2 protein–protein interactions, given marked obesity is a common finding in PWS.

Examining the interaction of NIPA1 protein with its 11 other interacting proteins indicates that three-fourths of the interactions are important for developmental bone morphogenesis or multifunctional proteins that control proliferation, differentiation, and other functions in many cell types (see Figure 3; Table 5). The interactions of NIPA2 protein with 19 other proteins indicate their collective role as intracellular signal transducers and transcriptional modulators that are activated by *TGFbeta,* and thereby impacting bone morphogenesis (see Figure 4; Table 8). CYFIP1 protein interacts with 25 other proteins. These proteins have a wide range of activity with functions including cytoskeleton organization and actin filament binding with cell-matrix adhesion, MAP kinase signal transduction impacts cell growth, survival and differentiation, stimulation of glucose uptake in cells, intracellular protein breakdown and tissue remodeling (see Figure 5; Table 11). TUBGCP5 protein interacts with 25 other proteins. These proteins are focused on mitotic spindle formation and assembly, microtubule organization, and production of centrosomal proteins involved in the regulation of centriole duplication during cell division (see Figure 6; Table 14).

### 3.1. Overlapping Association of PWS and AS Specific Genes with Both Syndromes at Varying Degrees

Although *UBE3A* is a classical AS gene (9.31 score), it is also associated with PWS to a lesser degree (4.106 score) (https://www.malacards.org/search/results/UBE3A). Further, it should be noted that the 15q11-q13 region includes the imprinted gene cluster with two maternally expressed protein-coding genes *UBE3A* and *ATP10A* (https://www.malacards.org/search/results?query=ATP10A). Similarly, all of the classical PWS genes overlap with both PWS and AS, albeit to varying degrees of significance: *MAGEL2*: PWS/AS: SCORE: 7.035/3.937; *NDN*: PWS/AS: SCORE: 7.435/4.528; *NDAP1*: PWS/AS: SCORE: 6.321/4.816; *SNRPN*: PWS/AS: SCORE: 6.563/5.584; *SNORD116*: PWS/AS: SCORE: 0.426/0.121; and even the IPW region: PWS/AS: SCORE: 7.761/0.100 (https://www.malacards.org). Therefore, it may not be appropriate to assume that PWS/AS are exclusively caused only by the designated PWS and AS regional genes per se, as shown in Figure 1, since most of these genes are also overlappingly associated with both PWS and AS, although to varying degrees. Moreover, PWS/AS phenotype is also overlappingly associated with three proximally located contiguous genes within the 15q11.2 BP1-BP2 region: *NIPA1, NIPA2*, and *CYFIP1*, as depicted in Table 16.

PWS is caused by loss of expression of the paternally inherited genes on chromosome 15q11.2-q13, and the cardinal features of PWS are attributable to the critical interval within the 15q11.2-q13 imprinted gene cluster, containing the small nucleolar RNA (snoRNA) *SNORD116* and non-coding RNA IPW (Imprinted in Prader–Willi) exons [39,40,41]. Similarly, the cardinal features of AS are attributable to the loss of expression of maternally inherited genes, and *UBE3A* in particular, which causes a distinct AS neurodevelopmental disorder [42].

Although the PWS core features are attributable to the critical interval within the 15q11.2-q13 imprinted gene cluster and the AS core features are attributable to the loss of expression of maternally inherited gene *UBE3A*, these core features might be influenced by several other genes that lie within the broader BP1-BP3 region. The functions of all of these genes are in-turn likely modulated by hundreds of their interacting genes that lie throughout the genome, as shown in the STRING protein interactions for *NAPI1, NAPI2, CYFIP1,* and *TUBGCP5:* Figure 2, Table 1; Figure 3, Table 5; Figure 4, Table 8; Figure 5, Table 11; Figure 6, Table 14; Table 15 and Table 16. One could speculate that PWS/AS, similar to that of ASD, depends on either the involvement of the PWS critical region in the imprinted segment within the 15q11.2-q13 BP2-BP3 region or the involvement of a critical gene, namely, UBE3A in the maternally imprinted segment [43] and in concert with other genes that lie within chromosome 15, not excluding the 15q11.2 BP1-BP2 region genes.

Every one of these genes along with their hundreds of interacting genes within the genome as a whole, as depicted and tabulated in this study pertaining to *NIPA1, NIPA2, CYFIP1,* and *TUBGCP5* genes all in concert could impact on the PWS/AS signature phenotype in any given proband. Thus, each PWS/AS proband’s clinical presentation is likely to be a unique genomic signature, within the broader spectrum of PWS/AS clinical presentations, which is akin to Autism Spectrum Disorder.

### 3.2. 15q11.2 B-P1-BP2 Microdeletion (Burnside–Butler) Syndrome: Characterization of Genes Within the BP1-BP2 Region, Meta-Analysis, and Parent-of-origin Effects Reveal Neurodevelopmental Associated Phenotypes

Each of the four evolutionarily conserved genes that escape imprinting and contiguously lie within the BP1-BP2 region are associated with distinct cardinal diseases: *NIPA1*: Spastic Paraplegia 6, Autosomal Dominant and Spastic Paraplegia 6; *NIPA2*: Angelman Syndrome and Prader–Willi Syndrome; *CYFIP1*: Fragile X Syndrome and Autism; and *TUBGCP5*: Prader–Willi Syndrome (see Table 15). Based on reports of their mutations or disturbed expression patterns, as reported in authentic web resources, such as, GeneCards.org, SAFARI.org, Gene Ontology (GO), OMIM.org, Entrez and UniProtKB/Swiss-Prot, their “collective loss or dosage duplication” is due to the BP1-BP2 microdeletion or microduplication that appears to impact only behavioral and neurological functions. These disorders include speech and motor delays, behavioral problems, seizures, and autism in affected individuals [44]. A recent review of the 15q11.2 BP1–BP2 microdeletion syndrome [1] found common phenotypic features, which included autism, developmental delay, motor and language delays, and behavioral problems.

Parental studies among these subjects demonstrated phenotypically normal carriers in several instances, and mildly affected carriers in others, complicating phenotypic association and/or causality. This could be due to either reduced penetrance or altered gene dosage on a particular genetic background [1,4]. The four non-imprinted and biallelically expressed genes, *NIPA1*, *NIPA2*, *CFYIP1* and *TUBGCP5*, in this microdeletion were initially noted to impact the severity of clinical presentation and neurological impairment in these two classical genomic imprinting disorders, Prader–Willi and Angelman syndromes with typical 15q11–q13 deletions depending on the absence or presence of the genomic area between breakpoints BP1 and BP2 containing these four genes, which led to the recognition of this microdeletion (*Burnside–Butler*) syndrome [4].

Therefore, it is intriguing to note that the review [4] of over 200 15q11.2 BP1–BP2 microdeletion syndrome individuals, as meta-analysis, did not find either the *NIPA1* associated cardinal disease: Spastic Paraplegia, the *NIPA2* associated cardinal diseases: Angelman Syndrome and Prader–Willi Syndrome, the *CYFIP1* associated cardinal disease: Fragile X Syndrome, or the *TUBGCP5* associated cardinal disease: Prader–Willi Syndrome, except for the *CYFIP1* associated cardinal disease: Autism, as presented in Table 15.

The clinical features as noted in the review [4] included developmental (73%) and speech (67%) delays; dysmorphic ears (46%) and palatal anomalies (46%); writing (60%) and reading (57%) difficulties, memory problems (60%) and verbal IQ scores ≤75 (50%); general behavioral, unspecified (55%) and abnormal brain imaging (43%). Other clinical features noted in this review [4], but not considered as common were seizures/epilepsy (26%), autism spectrum disorder (27%), attention deficit disorder (ADD)/attention deficit hyperactivity disorder (ADHD) (35%), schizophrenia/paranoid psychosis (20%), and motor delay (42%).

The review [4] also found that not all individuals with this deletion were clinically affected, but neuropsychiatric and behavior disturbances and mild dysmorphic features were associated with genomic imbalances of the 15q11.2 BP1–BP2 region, including microdeletions, but with an apparent incomplete penetrance and variable expressivity.

#### 3.3. All Four BP1-BP2 Region Genes Are Significantly Associated with Autism Spectrum Disorder

From reported patient cohorts presenting for genetic services and microarray analysis, this microdeletion syndrome can now be recognized as the most common cytogenetic abnormality found in ASD instead [6], which is not only a cardinal *CYFIP1* associated disease (see Table 15), but ASD is also consistently associated with *TUBGCP5, NIPA1,* and *NIPA2* genes (see Table 4, Table 7, Table 10, Table 13, and Table 16).

Thus, all four genes in this narrow segment between BP1 and BP2 are significantly associated with autism spectrum disorder, with an average of >68 MalaCards InFormaTion Score (MIFTS) with annotation strength (max 100) and with Solr (*an open-source enterprise-search platform*) relevance scores 0.171, 0.088, 4.578/0.377, and 0.21/0.094 for *NIPA1, NIPA2, CYFIP1,* and *TUBGCP5*, respectively (Table 4, Table 7, Table 10, Table 13, and Table 16). Moreover, even PWS and AS specific genes that lie in the BP2-BP3 PWS/AS region, such as, *MAGEL2, SNRPN, UBE3A,* and *ATP10A* are also significantly co-associated with autism (Malacards.org; SFARI.org) [45]. Therefore, it seems fair to conclude that except for PWS specific genes or transcripts, such as, snoRNAs, most other genes that flank on either side in broadly designated PWS and AS regions are all co-associated with autism. Most of the genes within the broader BP1-BP3 region are also co-associated with not only PWS and AS, but also with autism (Malacards.org; SFARI.org) [45]. Even the *GABRB3* gene that is distal to the *ATP10A* gene is a SFARI (SFARI.org) recognized autism gene (Malacards.org; SFARI.org) [45].

##### 3.4. 15q11.2 B–P1–BP2 Microdeletion (Burnside–Butler) Syndrome: Frequency

The 15q11.2 BP1–BP2 microdeletion syndrome has a reported de novo frequency between 5%–22%, with 51% having inherited the microdeletion from an apparently unaffected parent and 35% having inherited the microdeletion from an affected parent [4,6]. These low penetrance estimates may relate to subclinical manifestations of neuropsychiatric/behavioral problems, incomplete information, or lack of detailed clinical or psychiatric studies in the parents of individuals with 15q11.2 BP1–BP2 microdeletion or members of control cohorts.

###### 3.5. Summary

The four genes within the narrower proximal BP1-BP2 region, *NIPA1, NIPA2, CYFIP1,* and *TUBGCP5*, as well as those that lie within the broader BP2-BP3 region, such as *MAGEL2, SNRPN, UBE3A, ATP10A*, as well as *GABRB3* gene that are farther away from the distal *ATP10A* gene- are all recognized ASD genes. Furthermore, the 15q11.2 region collectively represents PWS, AS, and autism phenotypes (Malacards.org; SFARI.org) [45].

In addition to the above consistent correlation of each of the four genes that lie within the narrow proximal BP1-BP2 region with autism, in a more recent study [36] parent-of-origin effects (POE) of the 15q11.2 BP1-BP2 microdeletion were found to be associated with differences in clinical features in individuals inheriting the deletion. Among all probands studied, maternal deletions were found to be associated with epilepsy, autism spectrum disorder (*p* = 0.02) and macrocephaly (*p* = 0.016), while paternal deletions were associated with congenital heart disease (CHD) (*p* = 0.004) and abnormal muscular phenotypes (*p* < 0.05), while CHD and abnormal muscular phenotypes were seen in paternal deletions. This study not only supported POEs of this deletion, but notably included ASD, macrocephaly, and epilepsy.

The above key processes and summary of the four genes within the narrower proximal 15q11.2 BP1-BP2 region are collectively critical for normal neuronal development, plasticity, and function [1,5]. Their loss or gain (CNV) are associated with neurodevelopmental disorders, spastic paraplegia, seizures, learning, and gait disturbances with motor delay and neuro-behavioral psychiatric problems including autism, dyslexia, and schizophrenia/paranoid psychosis [1].

The most common maladies found to be individually associated with each of the four genes are: spastic paraplegia found in the top 12 of 15 disorders associated with *NIPA1*; PWS/AS, epilepsy and psychiatric/behavioral problems (autism or schizophrenia) in the top 10 disorders for *NIPA2*; fragile X syndrome, autism, schizophrenia, PWS and pervasive developmental disorder in the top 10 disorders for *CYFIP1*; and PWS, schizophrenia, autism, microcephaly, essential hypertension, body mass index (quantitative trait), epilepsy, and Down syndrome in the top 10 disorders associated with *TUBGCP5* (see Table 16). As noted in Table 15, all four genes are neither maternally nor paternally imprinted, but their proteins interact with each other as described and illustrated (see Figure 2, Table 1 and Table 2) in crucial biological processes (GO) and molecular pathways (GO). Collectively, either due to the microdeletion or microduplication, these genes predominantly affect brain morphology [1,4].

Our detailed exploration of the various aspects of each of these four genes, in solo and in concert is meant to enable an in-depth understanding of their individual and collective contribution in the causation of different neurodevelopmental phenotypes that have been variably reported in the literature. All four genes are individually associated with Prader–Willi syndrome, autism spectrum disorder, schizophrenia, epilepsy, and Down syndrome (Maladies.org; GeneCards.org). Except for the *TUBGCP5* gene, all three remaining genes are associated with Angelman syndrome, although the *TUBGCP5* gene is located most proximal to the Angelman syndrome gene, *UBE3A*. *TUBGCP5* gene is also not associated with attention deficit hyperactivity disorder and learning disability, developmental disorder, and peripheral nervous system disease. *CYFIP1* is the only gene that is not associated with microcephaly, but it is the only gene that is associated with a developmental disorder (see Table 16).

Thus, collectively, all four genes are associated with ten overlapping neurodevelopmental maladies up to 77.5% of the time.

## 4. Materials and Methods

We characterized the four genes (*NIPA1*, *NIPA2*, *CYFIP1,* and *TUBGCP5*) found within the 15q11.2 BP1-BP2 chromosome region of about 500 bp in size. When this region is deleted, an emerging disorder (i.e., Burnside–Butler syndrome) is being characterized leading to the importance of our description of these four genes. We undertook a literature review of hundreds of patients reported and summarized clinically with this microdeletion reported as the most common genetic defect found in consecutive patients presenting for genetic testing with high-resolution microarray analysis and autism spectrum disorder (e.g., [6]). Genome databases, search platforms for gene interaction, gene ontology (GO) biological process, molecular function, cellular components, along with KEGG and Reactome pathways using STRING network data along with sources, GeneCards.org, SAFARI.org, KEGG, PubMed, Gene Ontology (GO), OMIM.org, Entrez and UniProtKB/Swiss-Prot were summarized in tabular form and STRING Network results illustrated in figures and discussed in the text involving all four genes. An overview of each gene was also described throughout the summary process utilizing an approach reported previously [46].

**The Gene Ontology Resource** (**GO**; http://geneontology.org) provides structured, computable knowledge regarding the functions of genes and gene products. Founded in 1998, GO has become widely adopted in the life sciences, and its contents are under continual improvement, both in quantity and in quality. The Gene Ontology resource (GO; http://geneontology.org) is the most comprehensive and widely used knowledge base concerning the functions of genes. In GO, all functional knowledge is structured and represented in a form amenable to computational analysis, which is essential to support modern biological research. The GO knowledge base is structured using a formal ontology, by defining classes of gene functions (GO terms) developed over the past 20 years that have specified relations to each other [47]. GO terms include definitions, or equivalence axioms, that define the term relative to other terms in the GO or other ontologies. Their relationships can be computationally inferred using logical reasoning. The GO structure is constantly evolving in response to new scientific discoveries and are continuously refined using current biological information. The GO knowledgebase consists of ontology and annotations. As of the 5 September 2018 release (doi:10.5281/zenodo.1410625), there were ∼45000 terms in GO: 29698 biological processes, 11147 molecular functions, and 4201 cellular components, linked by almost 134000 relationships [47].

**MalaCards Relevance Scores** provide information used for the construction of our tables of Putative Associated Diseases (see Table 4, Table 7, Table 10 and Table 13) for the readership. MalaCards is an integrated database of human maladies and their annotations modeled from the architecture and information from the popular GeneCards database of human genes. The search platform used by MalaCards to obtain these data [48] is SOLR, an open-source full-text search platform widely used for analytics based on Apache’s Lucene text search API. When a term is searched by Lucene then it returns a set of scored hits. A “hit” represents a document (a MalaCard), whose fields (actual annotations) were previously indexed by Lucene.

The scoring is calculated by a Lucene defined algorithm:

score(q,d) = coord(q,d) · queryNorm(q) · ∑ tf(t in d) · idf(t) · t.getBoost() · norm(t,d) t in q

The factors in this formula are:

- tf stands for term frequency—the more times a search term appears in a document, the higher the score
- idf stands for inverse document frequency—matches on rarer terms count more than matches on common terms
- coord is the coordination factor—if there are multiple terms in a query, the more terms that match, the higher the score
- lengthNorm—matches on a smaller field score higher than matches on a larger field
- index-time boost—if a boost was specified for a document at index time, scores for searches that match that document will be boosted.
- query clause boost—a user may explicitly boost the contribution of one part of a query over another.

To ensure showing the best precision, MalaCards displays the score as (base 2 log of the score) + 10

For more about Lucene’s scoring mechanism see Apache Lucene—Scoring [48].

**GeneCards.org**—the human gene database [49] is from the GeneCards Suite: From Gene Data Mining to Disease Genome Sequence Analysis.

**STRING** protein network was utilized to generate Figure 2, Figure 3, Figure 4, Figure 5 and Figure 6 and their accompanying data on the protein–protein interactions as well as their Biological Processes (GO), Molecular Functions (GO), Cellular Component (GO) and KEGG and Reactome Pathways. The data are presented in Table 1, Table 2 and Table 3, Table 5, Table 6, Table 8, Table 9, Table 11, Table 12, and Table 14 and are adopted from STRING CONSORTIUM 2019. Data in Table 15 were derived from the GeneCards website (GeneCards.org) for respective genes, while the data for Table 16 were derived from MalaCards diseases (Malacards.org) as seen in Table 4, Table 7, Table 10, and Table 13 in this study.

## Figures and Tables

**Table 1:** STRING: Predicted Functions for *NIPA1*, *NIPA2*, *CYFIP1*, and *TUBGCP5* Genes.

GENE	Description	Categories							Score
***NIPA1***	**Magnesium transporter NIPA1; Acts as a Mg (2+) transporter. Can also transport other divalent cations such as Fe(2+), Sr(2+), Ba(2+), Mn(2+) and Co(2+) but to a much less extent than Mg(2+) (By similarity); Belongs to the NIPA family.**	***Gene Fusion***	***Co-occurrence***	***Co-expression***	***Experiments***	***Databases***	***Text mining***	***[Homology]***	
***TUBGCP5***	Gamma-tubulin complex component 5; Gamma-tubulin complex is necessary for microtubule nucleation at the centrosome.			**YES**			**YES**		**0.995**
***CYFIP1***	Cytoplasmic FMR1-interacting protein 1; Component of the CYFIP1-EIF4E-FMR1 complex which binds to the mRNA cap and mediates translational repression. In the CYFIP1-EIF4E-FMR1 complex this subunit is an adapter between EIF4E and FMR1. Promotes the translation repression activity of FMR1 in brain probably by mediating its association with EIF4E and mRNA (By similarity). Regulates formation of membrane ruffles and lamellipodia. Plays a role in axon outgrowth. Binds to F-actin but not to RNA. Part of the WAVE complex that regulates actin filament reorganization.			**YES**			**YES**		**0.967**
***NIPA2***	Magnesium transporter NIPA2; Acts as a selective Mg (2+) transporter; Belongs to the NIPA family.			**YES**			**YES**		**0.941**
***CFHR1***	Complement factor H-related protein 1; Involved in complement regulation.			**YES**					**0.937**
***SPG20***	Spartin; May be implicated in endosomal trafficking, or microtubule dynamics, or both. Participates in cytokinesis.						**YES**		**0.87**
***CFHR3***	Complement factor H-related protein 3; Might be involved in complement regulation.			**YES**					**0.832**
***SPAST***	Spastin; ATP-dependent microtubule severing protein that recognizes and cuts polyglutamylated microtubules.						**YES**		**0.808**
***MNS1***	Meiosis-specific nuclear structural protein 1; May play a role in the control of meiotic division and germ cell differentiation through regulation of pairing and recombination during meiosis.			**YES**					**0.769**
***IGFBP2***	Insulin-like growth factor-binding protein 2; Inhibits IGF-mediated growth and developmental rates.						**YES**		**0.746**
***BMPR2***	Bone morphogenetic protein receptor type 2						**YES**		**0.736**

Source: STRING.org.: *NIPA1*, *NIPA2*, *CYFIP1*, and *TUBGCP5*: STRING: 9606.ENSP00000337452.

**Table 2:** Predicted STRING Protein–Protein Functional Interactions for NIPA1, NIPA2, CYFIP1, and TUBGCP5 Genes.

GO-term	Description	Count in Gene Set	False Discovery Rate
	**Biological Process (GO)**		
GO:0061387	Regulation of extent of cell growth	3 of 96	0.0147
GO:1903830	Magnesium ion transmembrane transport	2 of 17	0.0179
GO:0050770	Regulation of axonogenesis	3 of 162	0.0179
GO:0001558	Regulation of cell growth	4 of 402	0.0179
GO:0120035	Regulation of plasma membrane-bounded cell projection organization	4 of 600	0.0243
GO:0045773	Positive regulation of axon extension	2 of 39	0.0243
GO:0090287	Regulation of cellular response to growth factor stimulus	3 of 254	0.025
GO:0048638	Regulation of developmental growth	3 of 302	0.0317
GO:0031346	Positive regulation of cell projection organization	3 of 343	0.0375
GO:0007052	Mitotic spindle organization	2 of 70	0.0375
GO:0030510	Regulation of BMP signaling pathway	2 of 86	0.0412
GO:0120034	Positive regulation of plasma membrane-bounded cell projection assembly	2 of 93	0.0456
	**Molecular Function (GO)**		
GO:0015095	Magnesium ion transmembrane transporter activity	2 of 17	0.0042

Source: STRING.org.: *NIPA1*, *NIPA2*, *CYFIP1*, and *TUBGCP5*: STRING: 9606.ENSP00000337452.

**Table 3:** STRING: Biological Processes (GO), Molecular Function (GO), Cellular Components with KEGG, and Reactome Pathway for the *NIPA1* Gene.

GO-term	Description	Count in Gene Set	False Discovery Rate
	**Biological Process (GO)**		
GO:0030509	BMP signaling pathway	10 of 92	3.30 × 10^−15^
GO:0010862	Positive regulation of pathway restricted SMAD protein phosphorylation	9 of 49	3.30 × 10^−15^
GO:0061448	Connective tissue development	11 of 194	7.18 × 10^−15^
GO:0030501	Positive regulation of bone mineralization	8 of 35	1.05 × 10^−14^
GO:0051216	Cartilage development	10 of 147	3.31 × 10^−15^
	**Molecular Function (GO)**		
GO:0005160	Transforming growth factor beta receptor binding	7 of 50	1.37 × 10^−11^
GO:0070700	BMP receptor binding	5 of 9	9.71 × 10^−11^
GO:0019199	Transmembrane receptor protein kinase activity	6 of 78	5.79 × 10^−9^
GO:0008083	Growth factor activity	7 of 160	5.79 × 10^−9^
GO:0005125	Cytokine activity	7 of 216	3.33 × 10^−8^
	**Cellular Component (GO)**		
GO:0043025	Neuronal cell body	6 of 460	0.00089
GO:1990712	HFE-transferrin receptor complex	2 of 8	0.0026
GO:0005615	Extracellular space	7 of 1134	0.0031
GO:0030425	Dendrite	5 of 531	0.0048
GO:0043235	Receptor complex	4 of 305	0.0052
	**KEGG Pathways**		
HSA04350	TGF-beta signaling pathway	9 of 83	5.28 × 10^−15^
HSA04390	Hippo signaling pathway	9 of 152	4.68 × 10^−13^
HSA04060	Cytokine–cytokine receptor interaction	6 of 263	3.07 × 10^−6^
HSA04360	Axon guidance	5 of 173	8.95 × 10^−6^
	**Reactome Pathways**		
HSA201451	Signaling by BMP	5 of 27	9.97 × 10^−9^
HAS9006936	Signaling by TGF-beta family members	6 of 100	3.10 × 10^−8^
HSA2129379	Molecules associated with elastic fibers	4 of 37	2.03 × 10^−6^
HSA1474244	Extracellular matrix organization	5 of 298	0.00017
HSA8866652	Synthesis of active ubiquitin: roles of E1 and E2 enzymes	2 of 30	0.0055

Source: STRING.org.

**Table 4:** Putative Diseases or Disorders Identifiable Using GeneCards.org, HGMD, and DISEASES as Referenced Sources for the *NIPA1* Gene.

Number	MalaCards ID	Name of Associated Disease	MIFTS	Solr Relevance Score
1	SPS127	Spastic Paraplegia 6, Autosomal Dominant	40	7.269
2	SPS041	Spastic Paraplegia 6	26	6.526
3	PRP016	Paraplegia	54	5.9
4	HRD010	Hereditary Spastic Paraplegia	67	5.563
5	ANG001	Angelman Syndrome	65	3.787
6	ATS013	Autosomal Recessive Congenital Ichthyosis	65	3.647
7	SPS215	Spastic Paraplegia 3, Autosomal Dominant	56	3.104
8	SPS147	Spastic Paraplegia 4, Autosomal Dominant	50	3.027
9	SPS148	Spastic Paraplegia 31, Autosomal Dominant	41	3.027
10	CMP101	Complex Hereditary Spastic Paraplegia	23	3.027
11	PRH002	Pure Hereditary Spastic Paraplegia	22	3.027
12	PRD006	Prader–Willi Syndrome	60	2.978
13	SPS107	Spastic Paraplegia 18, Autosomal Recessive	34	2.978
14	SPS099	Spastic Paraplegia 42, Autosomal Dominant	34	2.978
15	SPS021	Spastic Paraplegia 10	35	1.081
16	SCH015	Schizophrenia	76	0.187
17	ATS364	Autism	68	0.171
18	SPS057	Spasticity	38	0.171
19	AMY091	Amyotrophic Lateral Sclerosis 1	88	0.132
20	LTR001	Lateral Sclerosis	56	0.132
21	SPS012	Spastic Paraplegia 3a	26	0.132
22	EPL164	Epilepsy	73	0.108
23	HYP595	Hypertension- Essential	87	0.076
24	BDY004	Body Mass Index Quantitative Trait Locus 11	78	0.076
25	DWN001	Down Syndrome	70	0.076
26	ATS007	Autism Spectrum Disorder	69	0.076
27	THR014	Thrombocytopenia	67	0.076
28	DYS154	Dystonia	65	0.076
29	PRP019	Peripheral Nervous System Disease	64	0.076
30	PRC016	Pre-Eclampsia	63	0.076

MalaCards InFormaTion Score (MIFTS)—annotation strength (max 100). Solr Relevance Score is generated from the Apache Lucene project by integrating, indexing, and clustering data. Source: MalaCards.org, HGMD, OMIM, ClinVar, GTR, Orphanet, DISEASES, Novoseek, and GeneCards.org.

**Table 5:** STRING Interactants and Their Functions Related to Other Proteins Associated with NIPA1 Gene.

GENE	Description
***NIPA1***	Non-Imprinted in Prader-Willi/Angelman Syndrome Region Protein 1; Acts as a Mg (2+) transporter. Can also transport other divalent cations such as Fe (2+), Sr (2+), Ba (2+), Mn (2+) and Co (2+) but to a much less extent than Mg (2+) Belongs to the NIPA family.
***TGFB1***	Transforming growth factor beta 1; Multifunctional protein that controls proliferation, differentiation and other functions in many cell types.
***BMP4***	Bone morphogenetic protein- 4; Induces cartilage and bone formation. Also act in mesoderm induction, tooth development, limb formation and fracture repair.
***BMP6***	Bone morphogenetic protein 6; Induces cartilage and bone formation.
***BMP8B***	Bone morphogenetic protein 8B; Induces cartilage and bone formation. Plays a role in calcium regulation and bone homeostasis; Belongs to the TGF-beta family.
***MUSK***	Muscle, skeletal receptor tyrosine-protein kinase; Receptor tyrosine kinase which plays a central role in the formation and the maintenance of the neuromuscular junction (NMJ), the synapse between the motor neuron and the skeletal muscle.
***BMPR2***	Bone morphogenetic protein receptor type 2; On ligand binding, forms a receptor complex consisting of two type II and two type I transmembrane serine/threonine kinases.,
***BMP2***	Bone morphogenetic protein 2; Induces cartilage and bone formation. Stimulates the differentiation of myoblasts into osteoblasts via the EIF2AK3-EIF2A- ATF4 pathway
***BMP7***	Bone morphogenetic protein 7; Induces cartilage and bone formation. May be the osteoinductive factor responsible for the phenomenon of epithelial osteogenesis. Plays a role in calcium regulation and bone homeostasis.
***BMPR1B***	Bone morphogenetic protein receptor type-1B; On ligand binding, forms a receptor complex consisting of two type II and two type I transmembrane serine/threonine kinases.
***GDF2***	Growth/differentiation factor 2; Potent circulating inhibitor of angiogenesis. Signals through the type I activin receptor ACVRL1. Signaling through SMAD1 in endothelial cells requires TGF-beta coreceptor endoglin.

Source: STRING.org.

**Table 6:** Biological Process (GO), Molecular Function (GO), Cellular Component (GO) with KEGG, and Reactome Pathways for the *NIPA2* Gene.

GO-term	Description	Count in Gene Set	False Discovery Rate
	**Biological Process (GO)**		
GO:0007178	Transmembrane receptor protein serine/threonine kinase signaling pathway	16 of 189	1.49 × 10^−23^
GO:0030509	BMP signaling pathway	11 of 92	7.85 × 10^−17^
GO:0007167	Enzyme-linked receptor protein signaling pathway	17 of 698	7.85 × 10^−17^
GO:0010862	Positive regulation of pathway restricted SMAD protein phosphorylation	9 of 49	3.68 × 10^−15^
GO:0090100	Positive regulation of transmembrane receptor protein serine/threonine kinase signaling pathway	10 of 102	1.24 × 10^−14^
	**Molecular Function (GO)**		
GO:0046332	SMAD binding	9 of 73	6.30 × 10^−14^
GO:0004675	Transmembrane receptor protein serine/threonine kinase activity	7 of 17	6.30 × 10^−14^
GO:0005072	Transforming growth factor beta receptor, cytoplasmic mediator activity	6 of 10	9.18 × 10^−13^
GO:0019199	Transmembrane receptor protein kinase activity	8 of 78	3.47 × 10^−12^
GO:0030618	Transforming growth factor beta receptor, pathway-specific cytoplasmic mediator activity	5 of 5	1.79 × 10^−11^
	**Cellular Component (GO)**		
GO:0071141	SMAD protein complex	6 of 7	5.77 × 10^−13^
GO:0098802	Plasma membrane receptor complex	7 of 158	8.00 × 10^−8^
GO:0043235	Receptor complex	8 of 305	1.50 × 10^−7^
GO:1902554	Serine/threonine protein kinase complex	5 of 69	1.33 × 10^−6^
GO:0048179	Activin receptor complex	3 of 3	1.33 × 10^−6^
	**KEGG Pathways**		
hsa04350	TGF-beta signaling pathway	16 of 83	2.41 × 10^−30^
hsa04550	Signaling pathways regulating pluripotency of stem cells	12 of 138	2.17 × 10^−18^
hsa04390	Hippo signaling pathway	11 of 152	4.78 × 10^−16^
hsa04060	Cytokine-cytokine receptor interaction	7 of 263	4.15E × 10^−7^
	**Reactome Pathways**		
HSA9006936	Signaling by TGF-beta family members	12 of 100	2.36 × 10^−19^
HSA201451	Signaling by BMP	7 of 27	5.78 × 10^−13^
HSA1502540	Signaling by Activin	5 of 13	6.95 × 10^−10^
HSA181150	Signaling by NODAL	5 of 20	3.21 × 10^−9^
HSA3315487	SMAD2/3 MH2 Domain Mutants in Cancer	4 of 6	1.01 × 10^−8^

Source: STRING.org.

**Table 7:** Putative Associated Diseases for the *NIPA2* Gene.

Number	MalaCards ID	Name of Associated Diseases	MIFTS	Solr Relevance Score
**1**	PD006	Prader–Willi Syndrome	60	5.045
**2**	CHL002	Childhood Absence Epilepsy	60	4.53
**3**	ANG001	Angelman Syndrome	65	4.495
**4**	CHL058	Childhood Electroclinical Syndrome	21	3.568
**5**	ATS013	Autosomal Recessive Congenital Ichthyosis	65	2.523
**6**	SCH015	Schizophrenia	76	0.215
**7**	ATS364	Autism	68	0.196
**8**	EPL164	Epilepsy	73	0.124
**9**	HYP595	Hypertension, Essential	87	0.088
**10**	OST002	Osteoporosis	79	0.088
**11**	BDY004	Body Mass Index Quantitative Trait Locus 11	78	0.088
**12**	DWN001	Down Syndrome	70	0.088
**13**	ATS007	Autism Spectrum Disorder	69	0.088
**14**	THR014	Thrombocytopenia	67	0.088
**15**	MCR010	Microcephaly	56	0.088
**16**	BNM029	Bone Mineral Density Quantitative Trait Locus 15	51	0.088
**17**	HYD064	Hydrocephalus, Congenital, 1	47	0.088
**18**	BNM022	Bone Mineral Density Quantitative Trait Locus 8	43	0.088
**19**	CHR523	Chromosome 15q11.2 Deletion Syndrome	31	0.088
**20**	IMM162	Immunoglobulin E Concentration, Serum	29	0.088

MalaCards InFormaTion Score (MIFTS)-annotation strength (max 100). Source: MalaCards: HGMD, OMIM, ClinVar, GTR, Orphanet, DISEASES, Novoseek, GeneCards, and MalaCards.

**Table 8:** STRING Protein Interactants and Their Functions for *NIPA2.*

GENE	Description
***NIPA2***	Magnesium transporter NIPA2; Acts as a selective Mg(2+) transporter; Belongs to the NIPA family.
***ACVR1C***	Activin receptor type-1C; Serine/threonine protein kinase which forms a receptor complex on ligand binding.
***SMAD2***	Mothers against decapentaplegic homolog 2; also known as SMAD family member 2; Receptor-regulated SMAD (R-SMAD) that is an intracellular signal transducer and transcriptional modulator activated by TGF-beta (transforming growth factor) and activin type 1 receptor kinases.
***ACVR1***	Activin receptor type 1; On ligand binding, forms a receptor complex consisting of two type II and two type I transmembrane serine/threonine kinases.
***BMP6***	Bone morphogenetic protein 6; Induces cartilage and bone formation; Bone morphogenetic proteins.
***UXS1***	UDP-glucuronic acid decarboxylase 1; Catalyzes the NAD-dependent decarboxylation of UDP- glucuronic acid to UDP-xylose. Necessary for the biosynthesis of the core tetrasaccharide in glycosaminoglycan biosynthesis.
***SMAD3***	Mothers against decapentaplegic homolog 3; also known as SMAD family member 3; Receptor-regulated SMAD (R-SMAD) that is an intracellular signal transducer and transcriptional modulator activated by TGF-beta (transforming growth factor) and activin type 1 receptor kinases.
***SMAD4***	Mothers against decapentaplegic homolog 4; also known as SMAD family member 4; In muscle physiology, plays a central role in the balance between atrophy and hypertrophy.
***CHUK***	Inhibitor of nuclear factor kappa-B kinase subunit alpha; Serine kinase that plays an essential role in the NF- kappa-B signaling pathway.
***BMP5***	Bone morphogenetic protein 5; Induces cartilage and bone formation; Bone morphogenetic proteins.
***BMPR1A***	Bone morphogenetic protein receptor type-1A; On ligand binding, forms a receptor complex consisting of two type II and two type I transmembrane serine/threonine kinases.
***BMPR2***	Bone morphogenetic protein receptor type- 2; On ligand binding, forms a receptor complex consisting of two type II and two type I transmembrane serine/threonine kinases.
***TGFBR1***	TGF-beta receptor type-1; Transmembrane serine/threonine kinase forming with the TGF-beta type II serine/threonine kinase receptor. Regulating a plethora of physiological and pathological processes including cell cycle arrest in epithelial and hematopoietic cells, control of mesenchymal cell proliferation and differentiation, wound healing, extracellular matrix production, and immunosuppression.
***SMAD9***	Mothers against decapentaplegic homolog 9; also known as SMAD family member 9; Transcriptional modulator activated by BMP (bone morphogenetic proteins) type 1 receptor kinase.
***BMP7***	Bone morphogenetic protein 7; Induces cartilage and bone formation. May be the osteoinductive factor responsible for the phenomenon of epithelial osteogenesis. Plays a role in calcium regulation and bone homeostasis; Bone morphogenetic proteins.
***BMPR1B***	Bone morphogenetic protein receptor type-1B; On ligand binding, forms a receptor complex consisting of two type II and two type I transmembrane serine/threonine kinases.
***SMAD1***	Mothers against decapentaplegic homolog 1; also known as SMAD family member 1; Transcriptional modulator activated by BMP (bone morphogenetic proteins) type 1 receptor kinase.
***SMAD5***	Mothers against decapentaplegic homolog 5; also known as SMAD family member 5; Transcriptional modulator activated by BMP (bone morphogenetic proteins) type 1 receptor kinase. SMAD5 is a receptor-regulated SMAD (R-SMAD).
***ACVR1B***	Activin receptor type-1B; Transmembrane serine/threonine kinase activin type-1 receptor forming an activin receptor complex with activin receptor type-2 (ACVR2A or ACVR2B). Transduces the activin signal from the cell surface to the cytoplasm and is thus regulating many physiological and pathological processes including neuronal differentiation and neuronal survival, hair follicle development and cycling.

Source: STRING.org.

**Table 9:** STRING: Biological Process (GO), Molecular Function (GO), Cellular Component (GO) with KEGG, and Reactome Pathways for the *CYFIP1* Gene.

GO-term	Description	Count in Gene Set	False Discovery Rate
	**Biological Process (GO)**		
GO:0002252	Immune effector process	24 of 927	9.03 × 10^−27^
GO:0016192	Vesicle-mediated transport	25 of 1699	5.71 × 10^−23^
GO:0043312	Neutrophil degranulation	19 of 485	1.14 × 10^−22^
GO:0051179	Localization	26 of 5233	7.42 × 10^−14^
GO:0038096	Fc-gamma receptor signaling pathway involved in phagocytosis	8 of 73	4.66 × 10^−12^
	**Molecular Function (GO)**		
GO:0044877	Protein-containing complex binding	8 of 968	0.00062
GO:0008092	Cytoskeletal protein binding	8 of 882	0.00062
GO:0004252	Serine-type endopeptidase activity	5 of 180	0.00062
GO:0004175	Endopeptidase activity	6 of 399	0.00062
GO:0003779	Actin binding	6 of 413	0.00062
	**Cellular Component (GO)**		
GO:0035580	Specific granule lumen	14 of 62	9.87 × 10^−26^
GO:0034774	Secretory granule lumen	17 of 323	2.31 × 10^−22^
GO:1904724	Tertiary granule lumen	12 of 55	3.63 × 10^−22^
GO:0030141	Secretory granule	19 of 828	1.51 × 10^−19^
GO:0070820	Tertiary granule	13 of 164	4.33 × 10^−19^
	**KEGG Pathways**		
hsa04810	Regulation of actin cytoskeleton	10 of 205	1.08 × 10^−11^
hsa05131	Shigellosis	5 of 63	1.55 × 10^−6^
hsa04666	Fc gamma R-mediated phagocytosis	5 of 89	5.32 × 10^−6^
hsa04520	Adherens junction	4 of 71	7.99 × 10^−5^
	**Reactome Pathways**		
HSA168249	Innate Immune System	26 of 1012	9.01 × 10^−32^
HSA6798695	Neutrophil degranulation	19 of 471	8.77 × 10^−24^
HSA5663213	RHO GTPases Activate WASPs and WAVEs	10 of 35	3.26 × 10^−19^
HSA2029482	Regulation of actin dynamics for phagocytic cup formation	10 of 60	3.18 × 10^−17^
HSA4420097	VEGFA-VEGFR2 Pathway	7 of 95	1.15 × 10^−9^

Source: STRING.org.

**Table 10:** Putative Associated Diseases for *CYFIP1* Gene.

Number	MalaCards ID	Name of Associated Diseases	MIFTS	Solr
				Relevance
				Score
**1**	**FRG001**	**Fragile X Syndrome**	69	5.035
2	ATS364	Autism	68	4.578
3	SCH015	Schizophrenia	76	4.481
4	PRD006	Prader–Willi Syndrome	60	3.561
5	CHR523	Chromosome 15q11.2 Deletion Syndrome	31	3.561
6	PRV006	Pervasive Developmental Disorder	58	2.518
7	ATS007	Autism Spectrum Disorder	69	0.377
8	ALC028	Alacrima, Achalasia, and Mental Retardation Syndrome	65	0.188
9	HYP595	Hypertension, Essential	87	0.084
10	BDY004	Body Mass Index Quantitative Trait Locus 11	78	0.084
11	EPL164	Epilepsy	73	0.084
12	DWN001	Down Syndrome	70	0.084
13	LKM002	Leukemia	69	0.084
14	LKM062	Leukemia, Acute Lymphoblastic	68	0.084
15	NSP012	Nasopharyngeal Carcinoma	67	0.084
16	THR014	Thrombocytopenia	67	0.084
17	TRN020	Turner Syndrome	66	0.084
18	ANG001	Angelman Syndrome	65	0.084
19	ETN001	Eating Disorder	61	0.084
20	SQM006	Squamous Cell Carcinoma	60	0.084

MalaCards InFormaTion Score (MIFTS)-annotation strength (max 100). Source: MalaCards: HGMD, OMIM, ClinVar, GTR, Orphanet, DISEASES, Novoseek, and GeneCards.

**Table 11:** STRING Protein–Protein Interactants and Their Functions for *CYFIP1*.

GENE	Description
***CYFIP1***	Cytoplasmic FMR1-interacting protein 1; Component of the CYFIP1-EIF4E-FMR1 complex which binds to the mRNA cap and mediates translational repression. In the CYFIP1-EIF4E-FMR1 complex this subunit is an adapter between EIF4E and FMR1. Promotes the translation repression activity of FMR1 in brain probably by mediating its association with EIF4E and mRNA (By similarity). Regulates formation of membrane ruffles and lamellipodia. Plays a role in axon outgrowth. Binds to F-actin but not to RNA. Part of the WAVE complex that regulates actin filament reorganization.
***PGLYRP1***	Peptidoglycan recognition protein 1; Pattern receptor that binds to murein peptidoglycans (PGN) of Gram-positive bacteria. Has bactericidal activity towards Gram-positive bacteria.
***VCL***	Vinculin; Actin filament (F-actin)-binding protein involved in cell-matrix adhesion and cell-cell adhesion. Regulates cell- surface E-cadherin expression and potentiates mechanosensing by the E-cadherin complex. May also play important roles in cell morphology and locomotion; Belongs to the vinculin/alpha-catenin family.
***MAPK1***	Mitogen-activated protein kinase 1; Serine/threonine kinase which acts as an essential component of the MAP kinase signal transduction pathway. MAPK1/ERK2 and MAPK3/ERK1 are the 2 MAPKs which play an important role in the MAPK/ERK cascade. Depending on the cellular context, the MAPK/ERK cascade mediates diverse biological functions such as cell growth, adhesion, survival and differentiation through the regulation of transcription, translation, cytoskeletal rearrangements.
***CTSZ***	Cathepsin Z; Exhibits carboxy-monopeptidase as well as carboxy- dipeptidase activity.
***OLFM4***	Olfactomedin-4; May promote proliferation of pancreatic cancer cells by favoring the transition from the S to G2/M phase. In myeloid leukemic cell lines, inhibits cell growth and induces cell differentiation and apoptosis.
***CTSH***	Pro-cathepsin H; Important for the overall degradation of proteins in lysosomes; Belongs to the peptidase C1 family.
***RETN***	Resistin; Hormone that seems to suppress insulin ability to stimulate glucose uptake into adipose cells (By similarity). Potentially links obesity to diabetes (By similarity). Promotes chemotaxis in myeloid cells.
***ALDOC***	Aldolase, fructose-bisphosphate C.
***ARPC3***	Actin-related protein 2/3 complex subunit 3; Functions as component of the Arp2/3 complex which is involved in regulation of actin polymerization and together with an activating nucleation-promoting factor (NPF) mediates the formation of branched actin networks.
***LTF***	Lactotransferrin; Lactoferroxins A, B and C have opioid antagonist activity.
***CTSD***	Cathepsin D; Acid protease active in intracellular protein breakdown. Involved in the pathogenesis of several diseases such as breast cancer and possibly Alzheimer disease; Cathepsins.
***MMP8***	Neutrophil collagenase; Can degrade fibrillar type I, II, and III collagens; Belongs to the peptidase M10A family.
***WIPF3***	WAS/WASL-interacting protein family member 3; May be a regulator of cytoskeletal organization. May have a role in spermatogenesis (By similarity); Belongs to the verprolin family.
***TNFAIP6***	Tumor necrosis factor-inducible gene 6 protein; Possibly involved in cell-cell and cell-matrix interactions during inflammation and tumorigenesis.
***CFP***	Properdin; A positive regulator of the alternate pathway of complement. It binds to and stabilizes the C3- and C5-convertase enzyme complexes.
***CHI3L1***	Chitinase-3-like protein 1; Carbohydrate-binding lectin with a preference for chitin. Has no chitinase activity. May play a role in tissue remodeling and in the capacity of cells to respond to and cope with changes in their environment.
***DOCK2***	Dedicator of cytokinesis protein 2; Involved in cytoskeletal rearrangements required for lymphocyte migration in response of chemokines.
***TCN1***	Transcobalamin-1; Binds vitamin B12 with femtomolar affinity and protects it from the acidic environment of the stomach; Belongs to the eukaryotic cobalamin transport proteins family.
***ORM1***	Alpha-1-acid glycoprotein 1; Functions as transport protein in the blood stream. Binds various ligands in the interior of its beta-barrel domain. Appears to function in modulating the activity of the immune system during the acute-phase reaction.
***NCKAP1L***	Nck-associated protein 1-like; Essential hematopoietic-specific regulator of the actin cytoskeleton (Probable). Controls lymphocyte development, activation, proliferation and homeostasis, erythrocyte membrane stability, as well as phagocytosis and migration by neutrophils and macrophages. Component of the WAVE2 complex which signals downstream of RAC to stimulate F- actin polymerization.
***ABI2***	Abl interactor 2; May act in regulation of cell growth and transformation by interacting with nonreceptor tyrosine kinases ABL1 and/or ABL2. Part of the WAVE complex that regulates lamellipodia formation. The WAVE complex regulates actin filament reorganization via its interaction with the Arp2/3 complex.
***NCKAP1***	Nck-associated protein 1; Part of the WAVE complex that regulates lamellipodia formation. The WAVE complex regulates actin filament reorganization via its interaction with the Arp2/3 complex. As component of the WAVE1 complex, required for BDNF-NTRK2 endocytic trafficking and signaling from early endosomes.
***WASF1***	Wiskott-Aldrich syndrome protein family member 1; Downstream effector molecule involved in the transmission of signals from tyrosine kinase receptors and small GTPases to the actin cytoskeleton. Promotes formation of actin filaments. Part of the WAVE complex that regulates lamellipodia formation.
***BRK1***	Protein BRICK1; Involved in regulation of actin and microtubule organization. Part of a WAVE complex that activates the Arp2/3 complex. As component of the WAVE1 complex, required for BDNF- NTRK2 endocytic trafficking and signaling from early endosomes.
***WASF2***	Wiskott-Aldrich syndrome protein family member 2; Downstream effector molecule involved in the transmission of signals from tyrosine kinase receptors and small GTPases to the actin cytoskeleton. Promotes formation of actin filaments. Part of the WAVE complex that regulates lamellipodia formation. The WAVE complex regulates actin filament reorganization via its interaction with the Arp2/3 complex; Wiskott-Aldrich Syndrome protein family.

Source: STRING.org.

**Table 12:** STRING: Biological Process (GO), Molecular Functions (GO), Cellular Components (GO) with KEGG, and Reactome Pathways for the *TUBGCP5* Gene.

GO-term	Description	Count in Gene Set	False Discovery Rate
	**Biological Process (GO)**		
GO:0000086	G2/M transition of mitotic cell cycle	20 of 123	4.48 × 10^−36^
GO:1903047	Mitotic cell cycle process	25 of 564	2.99 × 10^−35^
GO:0010389	Regulation of G2/M transition of mitotic cell cycle	20 of 149	6.29 × 10^−35^
GO:0070925	Organelle assembly	25 of 666	6.97 × 10^−34^
	**Molecular Function (GO)**		
GO:0043015	Gamma-tubulin binding	6 of 28	3.66 × 10^−10^
GO:0015631	Tubulin binding	8 of 344	5.61 × 10^−7^
GO:0005200	Structural constituent of cytoskeleton	4 of 106	0.0004
GO:0008017	Microtubule binding	4 of 253	0.0065
GO:0030291	Protein serine/threonine kinase inhibitor activity	2 of 33	0.0155
	**Cellular Component (GO)**		
GO:0005813	Centrosome	23 of 468	3.98 × 10^−32^
GO:0005815	Microtubule organizing center	24 of 683	4.49 × 10^−31^
GO:0015630	Microtubule cytoskeleton	25 of 1118	1.64 × 10^−28^
GO:0044430	Cytoskeletal part	25 of 1547	3.73 × 10^−25^
GO:0044450	Microtubule organizing center part	14 of 167	5.27 × 10^−21^
	**KEGG Pathways**		
hsa04114	Oocyte meiosis	4 of 116	0.0013
hsa04110	Cell cycle	3 of 123	0.0218
hsa05203	Viral carcinogenesis	3 of 183	0.0447
hsa05169	Epstein–Barr virus infection	3 of 194	0.0447
	**Reactome Pathways**		
HSA380270	Recruitment of mitotic centrosome proteins and complexes	26 of 79	5.07 × 10^−59^
HSA380320	Recruitment of NuMA to mitotic centrosomes	26 of 91	4.11 × 10^−58^
HSA380259	Loss of Nlp from mitotic centrosomes	20 of 68	4.97 × 10^−42^
HSA8854518	AURKA Activation by TPX2	20 of 71	8.45 × 10^−42^
HSA2565942	Regulation of PLK1 Activity at G2/M Transition	20 of 85	1.69 × 10^−40^

Source: STRING.org.

**Table 13:** Putative Associated Diseases for the *TUBGCP5* Gene.

Number	MalaCards ID	Name of Associated Diseases	MIFTS	Solr Relevance Score
**1**	**PRD006**	**Prader–Willi Syndrome**	60	4.293
2	SCH015	Schizophrenia	76	0.23
3	ATS364	Autism	68	0.21
4	MCR010	Microcephaly	56	0.188
5	HYP595	Hypertension, Essential	87	0.094
6	BDY004	Body Mass Index Quantitative Trait Locus 11	78	0.094
7	EPL164	Epilepsy	73	0.094
8	DWN001	Down Syndrome	70	0.094
9	ATS007	Autism Spectrum Disorder	69	0.094
10	THR014	Thrombocytopenia	67	0.094
11	HYD064	Hydrocephalus, Congenital, 1	47	0.094
12	PRM031	Primary Autosomal Recessive Microcephaly	47	0.094
13	PRM212	Primary Microcephaly	42	0.094

MalaCards InFormaTion Score (MIFTS)-annotation strength (max 100). Source: MalaCards.Org: HGMD, OMIM, ClinVar, GTR, Orphanet, DISEASES, Novoseek, and GeneCards.

**Table 14:** STRING Interactants and Their Functions for the *TUBGCP5* Gene.

GENE	Description
*TUBGCP5*	Gamma-tubulin complex component 5; Gamma-tubulin complex is necessary for microtubule nucleation at the centrosome.
*HAUS4*	HAUS augmin-like complex subunit 4; Contributes to mitotic spindle assembly, maintenance of centrosome integrity and completion of cytokinesis.
*CEP41*	Centrosomal protein of 41 kDa; Required during ciliogenesis for tubulin glutamylation in cilium.
*HAUS3*	HAUS augmin-like complex subunit 3; Contributes to mitotic spindle assembly, maintenance of centrosome integrity and completion of cytokinesis.
*TUBGCP6*	Gamma-tubulin complex component 6; Gamma-tubulin complex is necessary for microtubule nucleation at the centrosome.
*HAUS8*	HAUS augmin-like complex subunit 8; Contributes to mitotic spindle assembly, maintenance of centrosome integrity and completion of cytokinesis.
*CEP135*	Centrosomal protein of 135 kDa; Centrosomal protein involved in centriole biogenesis. Acts as a scaffolding protein during early centriole biogenesis. Required for the targeting of centriole satellite proteins to centrosomes such as of PCM1, SSX2IP and CEP290 and recruitment of WRAP73 to centrioles. Also required for centriole-centriole cohesion during interphase.
*DCTN3*	Dynactin subunit 3; Together with dynein may be involved in spindle assembly and cytokinesis.
*HAUS2*	HAUS augmin-like complex subunit 2; Contributes to mitotic spindle assembly, maintenance of centrosome integrity and completion of cytokinesis.
*TUBGCP4*	Gamma-tubulin complex component 4; Gamma-tubulin complex is necessary for microtubule nucleation at the centrosome.
*TUBGCP3*	Gamma-tubulin complex component 3; Gamma-tubulin complex is necessary for microtubule nucleation at the centrosome.
*CEP76*	Centrosomal protein of 76 kDa; Centrosomal protein involved in regulation of centriole duplication. Required to limit centriole duplication to once per cell cycle by preventing centriole reduplication;
*CLASP1*	CLIP-associating protein 1; Microtubule plus-end tracking protein that promotes the stabilization of dynamic microtubules. Involved in the nucleation of noncentrosomal microtubules originating from the trans-Golgi network (TGN). Required for the polarization of the cytoplasmic microtubule arrays in migrating cells towards the leading edge of the cell.
*YWHAE*	14-3-3 protein epsilon; Adapter protein implicated in the regulation of a large spectrum of both general and specialized signaling pathways.
*CEP72*	Centrosomal protein of 72 kDa; Involved in the recruitment of key centrosomal proteins to the centrosome. Provides centrosomal microtubule-nucleation activity on the gamma-tubulin ring complexes (gamma-TuRCs) and has critical roles in forming a focused bipolar spindle, which is needed for proper tension generation between sister chromatids. Involved in centriole duplication.
*CEP70*	Centrosomal protein of 70 kDa; Plays a role in the organization of both preexisting and nascent microtubules in interphase cells. During mitosis, required for the organization and orientation of the mitotic spindle.
*PRKAR2B*	cAMP-dependent protein kinase type II-beta regulatory subunit; Regulatory subunit of the cAMP-dependent protein kinases involved in cAMP signaling in cells.
*PLK4*	Serine/threonine-protein kinase PLK4; Serine/threonine-protein kinase that plays a central role in centriole duplication. Able to trigger procentriole formation on the surface of the parental centriole cylinder.
*NINL*	Ninein-like protein; Involved in the microtubule organization in interphase cells. Overexpression induces the fragmentation of the Golgi and causes lysosomes to disperse toward the cell periphery; it also interferes with mitotic spindle assembly.
*CEP164*	Centrosomal protein of 164 kDa; Plays a role in microtubule organization and/or maintenance for the formation of primary cilia (PC), a microtubule-based structure that protrudes from the surface of epithelial cells. Plays a critical role in G2/M checkpoint and nuclear divisions. A key player in the DNA damage-activated ATR/ATM signaling cascade.
*MZT2B*	Mitotic spindle organizing protein 2B.
*HAUS1*	HAUS augmin-like complex subunit 1; Contributes to mitotic spindle assembly, maintenance of centrosome integrity and completion of cytokinesis.
*PLK1*	Serine/threonine-protein kinase PLK1; Serine/threonine-protein kinase that performs several important functions throughout M phase of the cell cycle, including the regulation of centrosome maturation and spindle assembly, the removal of cohesins from chromosome arms, the inactivation of anaphase-promoting complex/cyclosome (APC/C) inhibitors, and the regulation of mitotic exit and cytokinesis.
*YWHAG*	14-3-3 protein gamma; Adapter protein implicated in the regulation of a large spectrum of both general and specialized signaling pathways.
*PRKACA*	cAMP-dependent protein kinase catalytic subunit alpha; Phosphorylates a large number of substrates in the cytoplasm and the nucleus. Regulates the abundance of compartmentalized pools of its regulatory subunits through phosphorylation of PJA2 which binds and ubiquitinates these subunits, leading to their subsequent proteolysis. Required for glucose- mediated adipogenic differentiation increase and osteogenic differentiation inhibition from osteoblasts.
*MZT1*	Mitotic-spindle organizing protein 1; Required for gamma-tubulin complex recruitment to the centrosome.

Source: STRING.org.

**Table 15:** Summary of Functions, Nature, Compartmentalization, Related Pathways, and Cardinal Diseases Associated with *NIPA1*, *NIPA2*, *CYFIP1*, and *TUBGCP5* Genes in the 15q11.2 BP1-BP2 Region.

*NIPA1*	*NIPA2*	*CYFIP1*	*TUBGCP5*
**NIPA Magnesium Transporter 1**	**NIPA Magnesium Transporter 2**	**Cytoplasmic FMR1 Interacting Protein 1**	**Tubulin Gamma Complex Associated Protein 5**
***Protein Coding Gene***	***Protein Coding Gene***	***Protein Coding Gene***	***Protein Coding Gene***
**Cellular Compartmental Distribution with confidence number:**	**Cellular Compartmental Distribution with confidence number:**	**Cellular Compartmental Distribution with confidence number:**	**Cellular Compartmental Distribution with confidence number:**
plasma membrane (5) *	plasma membrane (4) *	extracellular (4) *	cytoskeleton (5) *
endosome (4) *	endosome (3) *	cytosol (4) *	cytosol (5) *
Golgi apparatus (1) *		cytoskeleton (1) *	nucleus (2) *
		mitochondrion (1) *	
		nucleus (1) *	
**Among its Related Pathways are:**	**Among its Related Pathways are:**	**Among its Related Pathways are:**	**Among its Related Pathways are: **
***miscellaneous transport and binding events and transport of glucose and other sugars, bile salts and organic acids, metal ions and amine compounds.***	***miscellaneous transport and binding events and transport of glucose and other sugars, bile salts and organic acids, metal ions and amine compounds.***	***Regulation of actin dynamics for phagocytic cup formation and signaling by Rho GTPases.***	***Nanog in Mammalian ESC Pluripotency and G-Beta Gamma Signaling.***
***Cardinal *Diseases Associated with *NIPA1*:**	***Cardinal* Diseases Associated with *NIPA2*:**	***Cardinal* Diseases Associated with *CYFIP1*:**	***Cardinal* Disease Associated with *TUBGCP5*:**
***Spastic Paraplegia 6, Autosomal Dominant & Spastic Paraplegia 6.***	***Angelman Syndrome and Prader–Willi Syndrome.***	***Fragile X Syndrome and Autism.***	***Prader–Willi Syndrome.***

Source: GeneCards.org, MalaCards.org, SAFARI.Org, KEGG, PubMed, Gene Ontology (GO), OMIM.org, Entrez, and UniProtKB/Swiss-Prot. * Subcellular Locations Confidence Levels: #5 denotes highest confidence level; #1 denotes lowest confidence level; Confidence Levels obtained from https://compartments.jensenlab.org.

**Table 16:** Summary of Associated Neurodevelopmental Maladies Across All Four Genes.

Number	Neurodevelopmental Disorders/Diseases	*NIPA1*	*NIPA2*	*CYFIP1*	*TUBGCP5*
1	Prader–Willi Syndrome	YES	YES	YES	YES
2	Angelman Syndrome	YES	YES	YES	NO
3	15q11.2 Deletion Syndrome with Attention Deficit Hyperactive Disorder & Learning Disability	YES	YES	YES	NO
4	Autism Spectrum Disorder	YES	YES	YES	YES
5	Schizophrenia	YES	YES	YES	YES
6	Epilepsy	YES	YES	YES	YES
7	Down Syndrome	YES	YES	YES	YES
8	Microcephaly	YES	YES	NO	YES
9	Developmental Disorder	NO	NO	YES	NO
10	Peripheral Nervous System Disease	YES	NO	NO	NO

Source: GeneCards.org, MalaCards.org, SAFARI.Org, KEGG, PubMed, Gene Ontology (GO), OMIM.org, Entrez. and UniProtKB/Swiss-Prot.
